# Detailed Characterization of Human Induced Pluripotent Stem Cells Manufactured for Therapeutic Applications

**DOI:** 10.1007/s12015-016-9662-8

**Published:** 2016-06-10

**Authors:** Behnam Ahmadian Baghbaderani, Adhikarla Syama, Renuka Sivapatham, Ying Pei, Odity Mukherjee, Thomas Fellner, Xianmin Zeng, Mahendra S. Rao

**Affiliations:** Lonza Walkersville, Inc., Walkersville, MD 21793 USA; Centre for Brain development and Repair, Institute of Stem Cells and Regenerative Medicine (InSTEM), Bangalore, India; Buck Institute for Researching on Aging, Novato, CA USA; XCell Science, Novato, CA USA; NxCell Inc, Novato, CA USA; Q therapeutics, Salt Lake City, UT USA

**Keywords:** Induced pluripotent stem cells, Embryonic stem cells, Manufacturing, cGMP, Consent, Markers

## Abstract

We have recently described manufacturing of human induced pluripotent stem cells (iPSC) master cell banks (MCB) generated by a clinically compliant process using cord blood as a starting material (Baghbaderani et al. in Stem Cell Reports, 5(4), 647–659, 2015). In this manuscript, we describe the detailed characterization of the two iPSC clones generated using this process, including whole genome sequencing (WGS), microarray, and comparative genomic hybridization (aCGH) single nucleotide polymorphism (SNP) analysis. We compare their profiles with a proposed calibration material and with a reporter subclone and lines made by a similar process from different donors. We believe that iPSCs are likely to be used to make multiple clinical products. We further believe that the lines used as input material will be used at different sites and, given their immortal status, will be used for many years or even decades. Therefore, it will be important to develop assays to monitor the state of the cells and their drift in culture. We suggest that a detailed characterization of the initial status of the cells, a comparison with some calibration material and the development of reporter sublcones will help determine which set of tests will be most useful in monitoring the cells and establishing criteria for discarding a line.

**Electronic supplementary material:**

The online version of this article (doi:10.1007/s12015-016-9662-8) contains supplementary material, which is available to authorized users.

## Introduction

Induced pluripotent stem cells (iPSCs) are akin to embryonic stem cells (ESC) [2] in their developmental potential, but differ from ESC in the starting cell used and the requirement of a set of proteins to induce pluripotency [3]. Although functionally identical, iPSCs may differ from ESC in subtle ways, including in their epigenetic profile, exposure to the environment, their mitochondrial content and perhaps X chromosome inactivation [4]. These differences are intrinsic to the source of starting material, and such differences may be further amplified by the pluripotency induction process [5]. It is important to note, however, that current studies have shown that these intrinsic differences between ESC and iPSC do not necessarily reflect in their functional utility; in fact, several large-scale analyses have verified that the differences seen are more reflective of the allelic diversity of individuals [6, 7]. The degree of difference seen between iPSC lines from different individuals is in the same range as differences seen between iPSC lines made from the same donor but different tissues and between ESC and iPSC [8, 9]. Equally important, the changes introduced by the process of iPSC generation using non-integration methods are in the same range as those changes that are seen when cells are maintained in culture for prolonged periods [10].

These differences, while of importance to the academic community, would be largely irrelevant to the regulatory authorities and for the development of an allogeneic or autologous product, provided these differences did not alter the potency or efficacy of the differentiated cells that were derived from these lines [11]. Indeed, this concept has been used for hematopoietic stem cell transplants where different donors (which differ in their allelic background and presumably in the efficacy) have been transplanted without showing that each sample was functionally identical by in vivo or in vitro testing [12]. It was presumed that the cells were functioning normally in the donor and that the harvesting process would not alter the cells (i.e. the cells were minimally manipulated), and one could reasonably infer that no change had occurred. This concept was extended to sibling or related transplants and then to matched unrelated donors (MUD) transplants. Here the inference was that one could reasonably extend the idea of functional equivalency even though the transplanted cells were functioning in a different setting.

This concept of functional equivalence was extended to cord blood, which is used for the same purpose as bone marrow but differs in that cord blood is processed differently from bone marrow and is considered more than minimally manipulated. The regulatory authorities reasoned that if functional equivalence in in vivo studies showed that cells could be manufactured reliably and reproducibly, then different groups using different processes and manufacturing at different sites could be approved under a Biologics License Application (BLA). Indeed, five public cord blood banks have been approved to provide MUD-type transplants for individuals using a commonly accepted release criteria for functional equivalence. Given the extent of in vivo human data available, no animal studies were required for the approval process. It is important to note that the regulatory authorities in the United States recognized that such licensure requirements should not be extended to autologous or related cord blood use, such as that proposed by private cord blood banks; indeed, those banks are not subject to the same BLA licensure requirements.

This logic has been extended to other autologous therapy where cells are more than minimally manipulated, such as autologous T-cells, B cells, dendritic cells, NK cells and macrophages [12]. Each of the cell populations is manufactured in a lot that is sufficient for one individual, and each lot is intrinsically different from another lot and is transplanted in a host at different stages of illness, where the cells likely encounter different environments. The authorities have not required that each lot undergo testing, as would be required for an allogeneic product that would be used for hundreds or thousands of patients. Rather, they have asked people to demonstrate that the end product obtained after processing is functionally equivalent [12]. In some cases the authorities have required that eight or ten samples manufactured be shown to be effective in an animal model, and in some cases have required human safety studies of a limited nature [12]. Critical to such approval has been the necessity of having adequate tests or comparability data that also assesses function [13].

We have assumed that the regulatory authorities will consider a similar logic for other autologous products or HLA-matched products, including those derived from iPSC [12]. Therefore, like cord blood, autologous or matched cells will be regulated differently than allogeneic products derived from iPSC. We further reasoned that if other groups wished to generate new lines they could use the same process and the community could define functional equivalence of these new lines. Since the lines themselves are merely input material to make fully differentiated cells, we felt that no animal tests were required at the iPSC stage; rather, criteria for use for further downstream processing could be established by in vitro differentiation assays and agreed-on quality control (QC) criteria for pluripotency. Functional characterization and equivalency of the end product with any necessary in vivo or human studies would occur on the final manufactured product. As with other products that may be used for autologous or allogeneic manufacture, we assume that the tests required will be different and the regulations likely different, but in both cases it will be critical to have comparability data.

Given most groups were initially focusing on allogeneic therapy, we initiated a program to generate clinically compliant cells and have reported on the generation of two such lines [1], which we presume will be used to generate a variety of products from a MCB. Although the process development was expensive and time consuming [1, 14], we reasoned that the cost would be amortized over a large number of patients [15]. Our data suggest that these lines could be used to commercialize iPSC-based cell therapy following a standard Investigational New Drug (IND) path.

However, it became evident that this was not a viable model for autologous cells and haplobank-derived cells, as had become clear with other cell therapies (see above). One would have to reduce cost and the regulators would need to develop models akin to those they have developed for cord blood banks. Therefore, we evaluated what would need to be done to reduce cost should cells be used for autologous therapy[13] or if a Haplobank was established[16]. Moreover, since these lines may be used by a number of individuals and utilized to generate a number of different products -- each of which will be likely manufactured in a different site by different companies -- we reasoned that additional characterization may be necessary and that a database to monitor changes in cells in culture needs to be established. In this manuscript, we describe the detailed characterization of two cGMP-compatible iPSC lines using WGS, array-based analysis and aCGH SNP analysis. One of these lines - LiPSC-GR1.1- generated during GMP manufacturing runs and the other line - LiPSC-ER2.2 - generated during engineering runs using the same GMP compatible process described before [1]. Our goal is to provide data to end users to determine which subset of tests will be required for ongoing monitoring, how such tests should be used to evaluate use of subclones for preclinical studies or cell therapy, and how comparability between manufacturing sites needs to be established.

## Materials and Methods

### Generation of Human Induced Pluripotent Stem Cells

Huamn iPSC lines used in the analysis and standard analysis (Table 1.0) were generated at Lonza Walkersville, Inc. as described before [1]. Briefly, cryopreserved human umbilical Cord Blood (hUCB) CD34^+^ cells (Lonza, 2C-101) were thawed and expanded in a priming medium comprised of a basal medium [including IMDM (Iscove's Modified Dulbecco's Medium; Life Technologies, 12440–053), Ham’s F12 (Life Technologies, 31765–035), Chemically Defined Lipid Concentrate (Life Technologies, 11905–031), Bovine Serum Albumin Fraction V (Life Technologies, 15260–037), and Insulin,-Transferrin-Selenium-Ethanolamine (ITS-X) (Life Technologies, 51500–056)] supplemented with 100 ng/mL recombinant human (rh)SCF (PeproTech, AF-300-07), 100 ng/ml rhFlt3-ligand (PeproTech, AF-300-19), 20 ng/ml rhThrombopoietin (PeproTech, 300–18) and 10 ng/ml IL-3 (PeproTech, 200–03). The CD34^+^ cells were seeded in 12-well plates (Corning, 3513) at a density of 4–6 × 10^5^ cells per well. Confluent cells (approximately day 3 post-thaw) were passaged the day prior to Nucleofection. 1 × 10^6^ hUCB CD34^+^ cells were reprogramed using the episomal plasmids encoding Oct4, Sox2, Klf4, c-Myc and Lin28 and pEB-Tg [17, 18]. These plasmids were introduced into the cells using the 4D-Nucleofector™ System and P3 solution Kit (Lonza, V4XP-3012). After nucleofection, the cells were plated in the priming medium in a 37 °C humidified incubator containing 5 % CO_2_ and 3 % O_2_. In some experimental conditions, thirty micrograms of Alhydrogel® adjuvant 2 % (InvivoGen, vac-alu-250) were immediately supplemented into the expansion medium to enhance the reprogramming efficiency. Two days post-plating, the cells were transferred into 6-well plates pre-coated with L7™ hPSC Matrix in the L7 hPSC medium supplemented with 1 μM TGFβ inhibitor (Stemgent, 04–0014). Cells were placed in a 37 °C humidified incubator containing 5 % CO_2_ and 3 % O_2_. The cell culture medium was changed every other day during the course of reprogramming step until hiPSC colonies appeared and were isolated for further expansion and characterization.

### Embryoid Body (EB) Differentiation

Confluent cultures of human pluripotent stem cell colonies were dissociated using L7™ hPSC Dissociation Solution. Cell aggregates were suspended in EB formation medium consisting of DMEM/F12 (Life Technologies, 11330–032) containing 10 μM Rock Inhibitor Y27632 (Millipore, SCM075) and allowed to settle by gravity in a conical tube. After removing the supernatant, cells were suspended in fresh EB medium. Cell aggregates were then seeded using a split ratio of 1:1 on Ultra Low Attachment (Corning, YO-01835-24) plates and returned to the incubator for 12 to 24 h. Once large cell aggregates formed, they were collected into a conical tube and allowed to settle by gravity. The medium was then removed and replaced with differentiation medium (80 % DMEM High Glucose (Life Technologies, 11965–092), 20 % defined fetal bovine serum (Hyclone, SH30070.03), 1X non-essential amino acids (Life Technologies, 11140–050), 2 mM L-glutamine (Cellgro/Mediatech, 25-005-CI) and 55 μM β-Mercaptoethanol (Life Technologies, 21985–023)). The cell aggregates were placed on Ultra Low Attachment plates using a split ratio of 1:1 in 0.4 ml differentiation medium/cm^2^. The culture medium was then changed every second day for six days. On the seventh day, EBs were seeded on gelatin-coated plates (EmbryoMax® ES Cell Qualified Gelatin Solution (Millipore, ES006-B)) at 10 EBs/cm^2^. The EBs were allowed to attach undisturbed for 2 days. The differentiation medium was changed after the second day and every other day afterward with 0.4 ml/cm^2^ differentiation medium. The cultures were prepared for immunocytochemistry on day 14.

Differentiated hPSCs were fixed with 4 % PFA and permeabilized with 0.1 % Triton X-100 PBS solution as described above. After rinsing the fixed cells with PBS-T solution, the cells were incubated with DPBS containing 10 % goat serum (Life Technologies, 10000C) for 2 h at room temperature. Primary antibodies detecting alpha-1 Fetoprotein (Abcam, ab3980; 1:200 or R&D systems, MAB1369, 1:100), beta III tubulin (Millipore, MAB1637; 1:400) and Smooth Muscle Actin (DAKO, M0851; 1:500) were added to blocked cultures and incubated overnight at 2–8 °C. The cells were rinsed twice with PBS-T, and the secondary antibody, Alexa Fluor 488-conjugated goat anti-mouse IgG(H + L) (Life Technologies, A11001; 1:1000) or Alexa Fluor 494-conjugated goat anti-mouse IgG(H + L) (Life Technologies, A-11032; 1:1000) were added and incubated on the cells for at least 2 h at room temperature. The cultures were then rinsed three times (5 min each) in 1X DPBS prior to counterstaining with DAPI. The cells were maintained in 50 % glycerol for analysis.

### Flow Cytometry

Flow cytometry of hPSCs was performed when cells reached approximately 70 to 80 % confluency in hPSC medium. The cultures were dissociated into a single-cell suspension using a solution of 0.05 % Trypsin/EDTA (CellGro, 25-052-CI) containing 2 % chick serum (Sigma-Aldrich, C5405). The cells were fixed and permeabilized for intracellular staining with the Cytofix/Cytoperm Kit (Becton Dickinson, 554714) following the manufacturer’s recommended protocol. Permeabilized cells were incubated with PE-conjugated anti-OCT3/4 (R&D Systems, IC1759P) or respective PE-conjugated anti-IgG isotype control. Extracellular antigens were detected on unfixed cells stained with PE-conjugated antigen-specific antibodies and respective isotypes using the manufacturer’s recommended concentration: anti-TRA-1-60 (Becton Dickinson, 560193), anti-TRA-1-81 (Becton Dickinson, 560161), anti-IgG3 isotype (Becton Dickinson, 556659); anti-SSEA4 (Becton Dickinson, 560128) and anti-IgM isotype (Becton Dickinson, 555584). The samples were then processed through a FACSCanto™ II flow cytometer (Becton Dickinson). Data were acquired using BD FACS Diva software and analyzed with Flowjo 7.6 software.

### Immunocytochemistry

Human pluripotent stem cells were cultured in the hPSC cell culture medium. hiPSC colonies present in the cultures on days 3 through 5 were prepared for immunocytochemical analysis as follows. The culture medium was aspirated, and cells were washed twice with 1X Dulbecco’s Phosphate Buffered Saline (Lonza Biosciences, 17-513 F). The cells were then fixed in 1X DPBS containing 4 % PFA (Electron Microscopy Sciences, 15710) for 20 min, then rinsed twice with PBS-T (0.2 % Tween-20 in 1X DPBS) for 5 min (Sigma-Aldrich, P9416), followed by a 2 h incubation with 10 % donkey serum in PBS-T at room temperature. The hPSCs were then treated with primary antibodies detecting extracellular antigens SSEA4 (Millipore, MAB4304; 1:100), TRA-1-60 (Millipore, MAB4360; 1:100) and TRA-1-81 (StemGent, 09–0011; 1:100) overnight at 2–8 °C prior to being permeabilized for 20 min in 1X DPBS containing 0.1 % Triton X-100 (Sigma-Aldrich, T9284). A second blocking step with 10 % donkey serum solution was performed before incubating the cells with intracellular primary antibodies overnight at 2–8 °C. Primary antibodies raised against pluripotency-associated antigens OCT4 (Abcam, ab19857; 1:350) and Nanog (R&D Systems, AF1997; 6.7 μg/ml) were used in combination with the secondary antibodies Cy3-conjugated Donkey anti-rabbit IgG (Jackson ImmunoResearch, 711-165-152; 1:200) and Cy3-conjugated donkey anti-Goat IgG (H + L) (Jackson ImmunoResearch, 805-165-180; 1:200), respectively. Primary antibodies specific for SSEA4 and TRA-1-60/TRA-1-81 were used in combination with secondary antibodies, Alexa Fluor 488-cojugated donkey anti-mouse IgG (H + L) (Jackson Immunoresearch, 715-545-150; 1:200) and Alexa Fluor 488-cojugated donkey anti-mouse IgM (H + L) (Jackson Immunoresearch, 715-545-140; 1:200), respectively. All cells were incubated with secondary antibodies for 2 h and then counterstained with 300 nM DAPI (Life Technologies, D3571) in 1X DPBS at room temperature for 15–30 min. Cells were rinsed after permeabilization and between the incubation of the primary and secondary antibodies. 50 % Glycerol was immediately added to the wells after the final wash with PBS-T. All fluorescence detection was visualized using an EVOS® FL all-in-one microscope equipped with software version 17625.

The immunocytochemistry and staining procedures of human pluripotent stem cells differentiated into neural lineage were as described previously [19]. Briefly, cells were fixed with 4 % paraformaldehyde for 20 min, blocked in blocking buffer (10 % goat serum, 1 % BSA, 0.1 % Triton X-100) for one hour, followed by incubation with the primary antibody at 4 °C overnight in 8 % goat serum, 1 % BSA, 0.1 % Triton X-100. Appropriately coupled secondary antibodies, Alexa350-, Alexa488-, Alexa546-, Alexa594- or Alexa633 (Molecular Probes, and Jackson ImmunoResearch Lab Inc., CA), were used for single or double labeling. All secondary antibodies were tested for cross reactivity and non-specific immunoreactivity.

### Expression Analysis by Microarray

Total RNA was isolated using the RNeasy® Mini kit according to the manufacturer’s instructions (Qiagen, CA) and hybridized to Illumina Human HT-12 BeadChip (Illumina, Inc., CA, performed by Microarray core facility at the Burnham Institute for Medical Research). All the data processing and analysis was performed using the algorithms included with the Illumina BeadStudio software. The background method was used for normalization. The maximum expression value of gene for probe set was used as the expression value of the gene. For the processed data, the dendrogram was represented by global array clustering of genes across all the experimental samples, using the complete linkage method and measuring the Euclidian distance. Expression of sample correlations was a measure of Pearson's coefficient, implemented in R System.

### CGH-CHIP Analysis

CGH-CHIP analysis was carried out using the aCGH + SNP service by Cell Line Genetics. Cryopreserved vials of the iPSCs were submitted to the contract lab to prepare sample and run the assay per standard procedures summarized below. The iPSC cryovials were thawed at 37 °C, washed once in 1xPBS, and centrifuged. The supernatant was then removed and the cell pellet was exposed to proteinase K and RNase and incubated at room temperature for two minutes. Following the addition of lysis buffer and incubation at 56 °C for 10 min, the samples were added to a DNeasy® mini spin column and attached by centrifugation. Samples were then washed two times with wash buffer and eluted in suspension buffer. gDNA samples were then cleaned using a Zymo DNA clean and concentration column. ChIP DNA binding buffer was added to the gDNA and added to a Zymo-Spin IC-XL column by centrifugation. The tube was washed two times with wash buffer and then eluted in DNA suspension buffer. DNA concentration and quality were determined using a NanoVue™ UV spectrophotometer, Qubit™ Fluorometer, and agarose gel analysis. The isolated gDNA must meet the following requirements: concentration of ≥1 μg of dsDNA measured by the Qubit™ Fluorometer; 260/280 Ratio of 1.76-1.9 measured by NanoVue™ Spectrophotometer; and 260/230 Ratio of ≥1.9 measured by NanoVue™ Spectrophotometer.

Following gDNA isolation, labeling reactions were prepared using the Agilent SureTag Complete Labeling Protocol for aCGH with 500–1500 ng total (RNase treated) DNA input. The Agilent microarray aCGH protocol composed of two steps: Labeling of the DNA and hybridization. First, equal amounts of both test and reference samples (500–1500 ng) were enzymatically sheared for aCGH + SNP arrays using a dual DNA digestion with restriction enzymes *Rsa1* and *Alu1.* The test sample DNA was labeled with Cyanine 5-dUTP and the reference DNA was labeled with Cyanine 3-dUTP by Exo-Klenow fragment. The labeled DNA was then purified, and the labeling efficiency and concentration were determined using the NanoVue™ UV spec. The test and appropriate reference samples were then combined and denatured. The labeled probes were allowed to hybridize with the feature on the microarray for 24 h at 65 °C. Finally, the arrays were stringently washed and scanned at a 3 μM resolution on an Agilent SureScan Microarray Scanner. Feature data was extracted, processed and mapped to the human genome (hg19) using ADM-2 Segmentation Algorithm using Agilent CytoGenomics.

### Whole Genome Sequencing

Whole genome sequencing was performed by Macrogen Clinical Laboratory (Rockville, MD). The samples were prepared according to an Illumina TruSeq Nano DNA sample preparation guide. Briefly, the whole genomic DNA was extracted using the DNeasy® Blood & Tissue Kit according to manufacturer’s instructions (Qiagen, CA cat#69506). One microgram of genomic DNA was then processed using the Illumina TruSeq DNA PCR-Free Library Preparation Kit to generate a final library of 300–400 bp fragment size. Completed, indexed library pools were run on the Illumina HiSeq platform as paired-end 2x150bp runs. FASTQ files were generated by bcl2fastq2 (version 2.15.0.4) and aligned by ISAAC Aligner (version 1.14.08.28) to generate BAM files. SNPs, Indels, structural variants (SV) and copy number variants (CNV) were detected by ISAAC Variant Caller version 1.0.6 [20]. For the SNPs and Indel, locus reads with genotype quality less than 30 were removed from analysis. The vcf file thus generated was annotated using SNPEff Version 4.0e (http://snpeff.sourceforge.net/) [21] using hg19 reference genome, dbSNP138 build. The alternate allele frequency for European descendent samples were obtained from 1000 genome project_phase1_release_v3 and ESP6500 databases.

Samtools was used to obtain basic statistics such as the number of reads, number of duplicate reads, total reads mapped and total reads unmapped. SAMSTAT version 1.5.1 (http://samstat.sourceforge.net/) [22] was used to report the mapping quality statistics. The depth of each chromosome was computed by Issac variant caller.

The variants derived were used to predict the blood group phenotypes, with the analytical software Boogie [23]. Blood group predictions were made for routinely used ABO and Rh system. Apart from this, predictions for MN- and Rh-associated glycoprotein systems were also performed for both the cell lines. Genotype information including the chromosome number, genomic position, reference allele, alternate allele and zygosity of the variants belonging to the genes involved in the above mentioned blood group systems were provided as an input. Boogie verified the relevant variants in the input genotype with defined phenotypes in the haplotype table provided default by the software, based on 1-nearest neighbor algorithm. The SNV permutation with the most likely phenotype gets the best score. The blood groups thus predicted were compared with available donor data.

The HLA class I (HLA-A,-B and -C) and II (HLA-DQA1, −DQB1 and -DRB1) profiles for the iPSC lines were estimated from the WGS data by software called HLAVBseq, which was developed by Nariai and colleagues [24]. FASTQ files were aligned to the reference genome using BWA-MEM to generate a *sam* file. This method is based on the alignment of sequence reads to the genomic HLA sequences that are registered with IMGT/HLA database. Based on variational Bayesian inference statistical framework, the expected read counts on HLA alleles is estimated. The hyper parameter alpha zero for paired end data set to 0.01. The average depth of coverage for each HLA allele was calculated based on the perl script provided by the authors for 200 bp data. The predicted HLA types was cross-verified with HLA typing results generated by HLAssure^TM^ SE SBT kit.

To verify if these cell lines showed any variations in genes implicated in PD, only the non-synonymous variants were considered. The variants were prioritized based on the benign or damaging effect of the amino acid substitutions. The *in-silico* predictions program, such as SIFT, bases its predictions on the degree of conservation of amino acid residues [25], whereas Polyphen predicts these changes based on the impact of the amino substitution on the structure and function of the protein based on physical and comparative considerations [26], respectively. The scores of SIFT and Polyphen were computed by dbNSFP[27]. The prioritized variants were cross-validated with the list of PD related genes obtained from gene cards (www.genecards.org). The variants shortlisted were referred to clinvar and MIM_disease databases, annotated by dbNSFP. The integrative study of WGS and expression data was performed to validate if these PD-related genes showed any variation in their expression against the control lines H9, H7 and NCRM6 [28].

The Issac variant caller which was used to detect structural variants for the cell lines showed highest number of deletions, and hence were considered for the analysis. The variants with genotype quality <20, reads with MAPQ of zero around either break-end or unknown exact breakpoint location, and read pairs that support the variant with low confidence were removed. The filtered variants were annotated for the genes using UCSC table browser (https://genome.ucsc.edu/cgi-bin/hgTables). Deletion events were manually viewed by Integrated Genomics Viewer (IGV) [29] to check for the dip in the coverage at deletion sites. Bed Tools v2-2.20.1 was used to check the level of overlap from two sets of genomic coordinates. This data was cross-validated with expression data to verify if there was any differential expression levels due to these deletions. These short listed differentially expressed genes were checked for gene enrichment to verify their implications in various OMIM_diseases and pathways [30]. A similar strategy was followed for duplications [31]. The results were compared with SNP CHIP data for both the cell lines to check for the gene overlap, if any. This cross comparison was made even with the unfiltered SV data, to verify if the genes identified by micro array were missed in WGS due to filtering.

To verify the status of the imprinted genes, the published list of imprinted genes was extracted from the database (http://www.geneimprint.com/). Allelic depth of the alternate allele, if <10, was filtered. The number of heterozygous and homozygous SNPs, INDELS were calculated for these imprinted genes and verified for the genes overlap with expression data. The maternal or paternal specific expression for these genes were reported from the documented data. As no parental information was available, phasing could not be conducted on these samples to identify maternal or paternal specific inheritance pattern of these variants identified by the WGS.

### HLA Type Analysis

HLA-typing was carried out by Texas Biogene, Inc. (Richardson, TX) using HLAssure^TM^ SE SBT typing kits. The HLAssure^TM^ SE SBT Kit is for determining HLA alleles using PCR amplification with sequence based typing (PCR-SBT) methodology. Briefly, the whole genomic DNA was extracted using the DNeasy® Blood & Tissue Kit according to the manufacturer instructions (Qiagen, CA cat#69506) and per requirements suggested by Texas Biogene, Inc. (i.e. DNA sample with an A260/A280 ratio between 1.65 and 1.8). The genomic DNA was then analyzed using the HLAssure^TM^ A, B, C, DRB1, and DQB1 SBT typing kits and Accutype^TM^ (HLADB-3.19.0) software per procedure established by Texas Biogene, Inc.

### Karyotype and Short Tandem Repeat (STR)

Karyotype and STR analyses were performed by a qualified service provider (Cell Line Genetics) using standard methods. Human G-banding karyotyping was performed in accordance with FDA Good Laboratory Practice by Cell Line Genetics, which was audited by Lonza Walkersville, Inc. with clinically certified cytogeneticists experienced with identifying chromosomal abnormalities from pluripotent stem cells. For each cell line, 20 chromosomes were analyzed from live or fixed cells in metaphase. The analysis was performed using G-banding and Leishman stain, and the Cells were analyzed according to the Clinical Cytogenetics Standards and Guidelines published by the American College of Medical Genetics [32].

The STR assay utilized PCR and capillary electrophoresis on a PowerPlex 16 mutiplex STR platform (Promega) to determine a match of ≥80 % of the 16 loci evaluated. Data analysis was performed with SoftGenetics Genemarker software. Each assay was evaluated for off ladder peaks, considered artifacts, and cross contamination prior to reporting.

### MCB Viral Testing

According to FDA regulations, release of allogeneic MCBs for clinical use requires extensive testing for the presence of viral contaminates. The scope of the MCB viral testing for hiPSCs was adjusted based on the cellular characteristics of pluripotent stem cells and comprised of both in vitro and in vivo assays [1]. Following preparation of samples per standard procedures recommended by the contract lab (BioReliance), samples were submitted to the BioReliance in appropriate condition and format. BioReliance is fully accredited for GLP, and all studies conducted by BioReliance are performed in compliance with the requirements of the UK and German GLP Regulations, the US FDA Good Laboratory Practice Regulations (21 CFR 58), the Japanese GLP Standard, and the OECD Principles of Good Laboratory Practice (http://www.bioreliance.com/us/about-us).

### Assay Qualification, Characterizations, and in Process Control

#### Flow Cytometry Assay for Pluripotent Stem Cells

The flow cytometry assay for evaluation of human pluripotent stem cells was qualified according to the current Good Manufacturing Practices, the International Conference on Harmonization Technical Requirements for Registration of Pharmaceuticals for Human Use (ICH) validation guidelines [33]. The qualification study was conducted using stage-specific embryonic antigen-4 (SSEA-4), Tra-1-60, and Tra-1-81. In addition, Oct4, a transcription factor thought to play a key role in maintaining the self-renewal and pluripotency of the embryonic stem cells [34], was also included in the qualification study. The release criteria for pluripotency markers were established based on the positive expression of four different markers (SSEA-4, Tra-1-60, Tra-1-81, and Oct3/4) according to published data as well as data generated during the process development phase. Since cord blood derived CD34 positive cells were utilized as a starting material for reprogramming and generation of the final product hiPSCs, negative expression of surface marker CD34 was also included in the qualification study. Precision (intra-assay, inter-assay, and intermediate), accuracy, specificity and sensitivity of this flow cytometry assay was determined during the qualification study. The qualified flow cytometry assay with established release criteria was later used to evaluate the purity and identify of human iPSCs.

#### Quantitative PCR for Evaluation of Residual Plasmid Clearance

Since human induced pluripotent stem cells (iPSCs) were generated using pEB-C5 (i.e. an EBNA1/OriP episomal plasmid expressing Oct4, Sox2, Klf4, c-Myc and Lin28) and pEB-Tg (i.e. an EBNA1/OriP plasmid for transient expression of SV40 T antigen), a quantitative PCR (qPCR) assay (“Residual qPCR”) was developed to quantitatively detect residual EBNA/OriP sequences originating from either pEB-C5 or pEB-Tg. Both pEB-C5 and pEB-Tg are non-integrating plasmids that are supposed to become clear following serially passaging of hiPSCs [18, 35]. Considering the goal of assay to determine the clinical safety of the hiPSC clones generated by episomal plasmids, the Residual qPCR assay was qualified according to the International Conference on Harmonization Technical Requirements for Registration of Pharmaceuticals for Human Use (ICH) validation guidelines. Accuracy, specificity, limit of detection (LOD), and limit of quantification (LOQ) were determined during the PCR qualification studies. This qualification study was executed based on 9 total assays conducted by 3 analysts on 3 separate days using a validated qPCR machine. Appropriate control positive, control negative, and reference materials were used in the qualification study.

#### Characterization Assays

Evaluation of hiPSC colony morphology, plating efficiency of hiPSCs post-thaw, and embryoid body (EB) formation were classified as FIO assays due to the challenges associated with qualification of these assays, in particular the subjective interpretation of the results. EB formation was used to demonstrate the identity and potency of hiPSCs by investigating spontaneous differentiation into three germ layers (i.e. ectoderm, mesoderm, and endoderm) and evaluating the results through immunofluorescence at the protein level or qPCR analysis at the transcript level. Post-thaw plating efficiency was evaluated based on alkaline phosphatase (AP) staining. AP, a hydrolase enzyme responsible for dephosphorylating molecules such as nucleotides, proteins, and alkaloids under alkaline conditions, has been widely used for evaluation of undifferentiated pluripotent stem cells, including both embryonic stem cells and iPSCs [3, 36–38]. Upon staining, the undifferentiated cells appear red or purple, whereas the differentiated cells appear colorless. However, considering the inconsistencies observed in the quality of AP reagents offered by different suppliers and subsequent intensity of AP staining, it was difficult to set specifications and cut-off values for the cells positively stained with AP marker, which in turn resulted in inability to qualify this assay.

### Pluritest

Pluritest is an online bioinformatic assay based on gene expression collected from Illumina microarray to verify pluripotency [3, 39]. Pluritest is based on 450 genome-wide transcriptional profiles. These samples are from multiple laboratories and vary from diverse stem cell samples to differentiated cell types, developing and adult human tissue. 223 samples are human embryonic stem cells and 41 are from iPSC’s. Two models were developed to obtain pluripotency and non-pluripotency, a pluripotency score and a novelty score. Pluripotency score is based on expression levels from known pluripotent and non-pluripotent genes in the 450 genome-wide transcriptional profiles. Unknown samples’ gene expression levels are compared to the expression levels from the 450 samples, and their pluripotency is based on this comparison. Novelty score measures the technical and biological variation, based on comparing samples to well-known PSC s in the dataset [40].

### In Process Assays

One important aspect of the GMP manufacturing process was to establish appropriate assays at different stages of the process to ensure the quality of intermediate materials and monitor the progress of the process during the long manufacturing process. For instance, a flow cytometry assay was implemented following the isolation of CD34+ cells but before proceeding with the priming step. This step ensured appropriate population of actively proliferating CD34+ cells (i.e. a minimum of 40 % CD34+ cells) were included in the expansion phase (priming step) prior to the reprogramming (Nucleofection) step. Importantly, the selection of best hiPSC colonies for expansion was based on the quality of hiPSCs observed during the expansion phase as well as level of residual plasmid present in the samples taken from each hiPSC clone. A scoring system was established to evaluate the quality of hiPSC cultures after isolation and throughout the course of serial subculturing of the cells based on the attachment of hiPSC colonies the day after passaging, evaluation of the confluency and amount of spontaneous differentiation at each passage and every day, and elapsed time (days) per passage. Following selection and establishing the hiPSC clones, in-process samples were submitted at the end of each passage to detect the level of residual plasmids (i.e. residual EBNA/OriP sequences), using qPCR analysis and by recording the Ct value. The Ct values and the scores achieved for each clone were used to evaluate the best clone(s) to use in further manufacturing, scale-up and banking process.

## Results

### Basic Cell Line Characterization

**Fig. 1:**
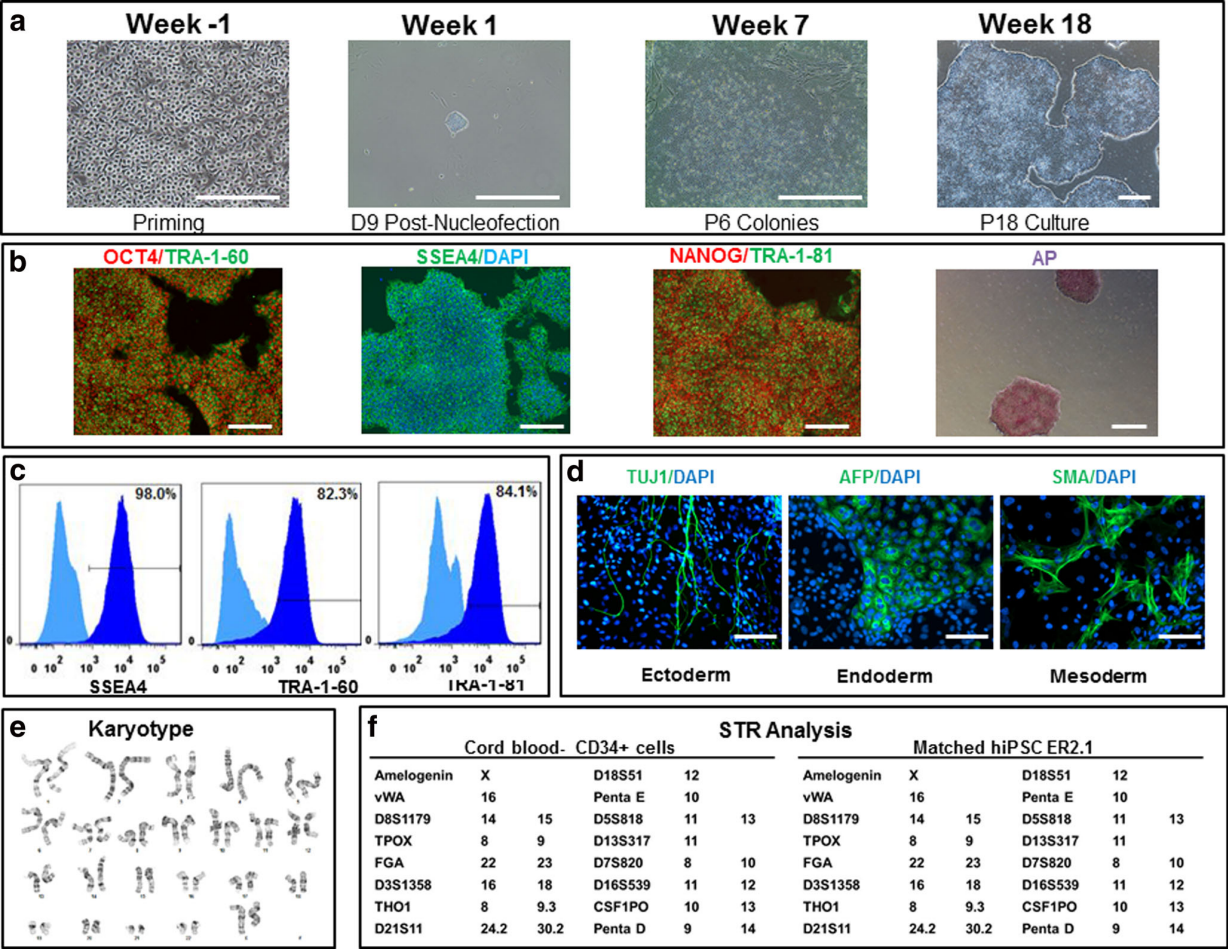
Generation, expansion, and characterization of human iPSCs (LiPSC-ER2.1) - engineering runs. Panel **a** illustrates priming of CD34+ Cells isolated from cord blood unit and expanded in culture on day 3 prior to the nucleofection (Priming), iPSC colony emerged on day 9 post nucleofection (D9 Post-Nucleofection), iPSCs at passage 6 (P6 Colonies), and iPSCs at passage 18 (P18 culture). Panel **b** illustrates iPSCs positively stained with OCT4, TRA-1-60, SSEA4, NANOG, TRA-1-81, and alkaline phosphatase (AP). Panel **c** shows the iPSCs expressing the pluripotent stem cell surface markers SSEA4, TRA-1-60, and TRA-1-81 (*dark blue*). Light blue exhibits the isotype control. Panel **d** shows iPSCs differentiated into embryoid bodies and readily expressed the markers for early ectoderm (TUJ1), endoderm (Alpha-Feto Protein (AFP)), and mesoderm (Smooth Muscle Actin (SMA)). DAPI shows the nuclei stain in blue. The iPSCs demonstrated normal karyotype after 17 passages (**e**). STR analysis showed that the iPSCs matched the starting CD34+ donor sample (**f**). *Scale bar* in all images in Panel **a** is 500 microns except the Priming image which is 250 microns. *Scale bar* in all images in Panel **b** is 250 microns except the AP image which is 500 microns. *Scale bar* in all images in Panel D is 125 microns

**Fig. 2:**
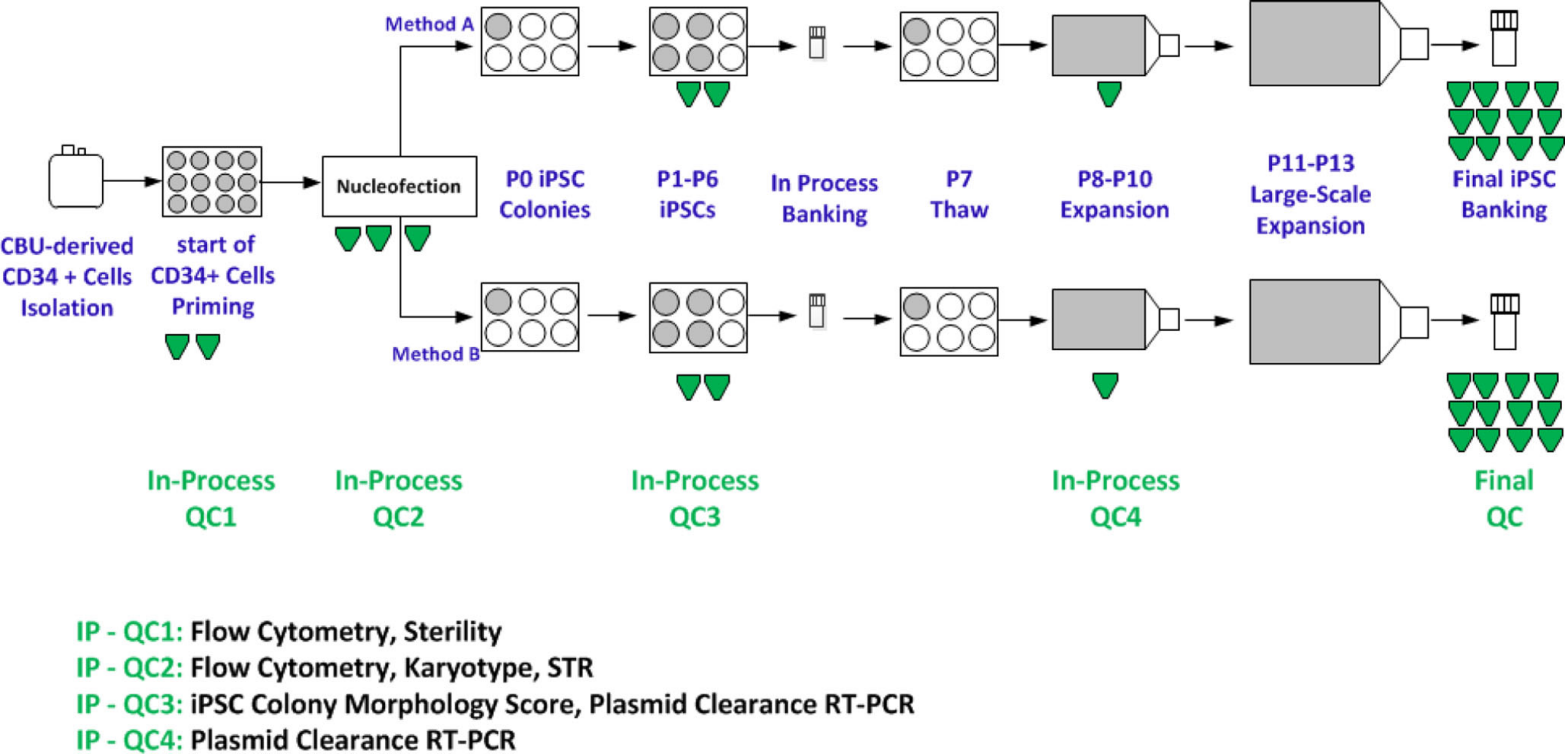
Human iPSC manufacturing process flow diagram with in process testing of samples. The process for manufacturing of human iPSCs under defined and cGMP conditions include (1) isolation of CD34+ cells from fresh cord blood unit, (2) priming CD34+ cells for 4 days, (3) reprogramming of CD34+ cells into iPSCs using 4D Nucleofector system and an episomal based technology, (4) isolation of about 9 iPSC colonies, serial subculturing of iPSCs up to 6 passages, (5) in process cryopreservation of all iPSC colonies to select the two best iPSC colonies based on the results of in process control IP-QC3, (6) expansion of two selected iPSC colonies and confirmation of plasmid clearance, (7) further expansion into large tissue culture flasks, (8) banking, and (9) final characterizations and QC testing. Multiple in-process samples (shown in *green*) were taken at different stages of the process and submitted for relevant testing. Each in-process test has been described in the figure. The number of in-process samples exhibits the number of tests carried out at each step

As we developed a process for cGMP manufacture of hiPSCs [1, 14], we have generated over fifty lines using different methods and numerous donors to evaluate an optimal method that worked reliably and reproducibly in our hands. During the process development, we identified a critical set of required tests (Table 1) that were similar to those required for most of the cell lines. In addition, we added karyotype analysis as a key release assay, because karyotype abnormalities (e.g. trisomies of chromosomes 12 and 17) have been observed with in vitro cultures of both human ESCs [41, 42] and iPSCs [43], and these abnormalities are suggested to be characteristics of malignant germ cell tumors [44, 45]. As no required tests have been defined by regulatory authorities on determining the quality of ESC or iPSC lines, we reasoned that determining performance of the lines based on their use would be a reasonable start. We determined that pluripotency could be determined by the presence of pluripotency markers that were assessed by immunocytochemistry using well characterized and widely accepted markers (Fig. 1), and by performing functional assays on the ability of the cells to differentiate into ectoderm, endoderm and mesoderm (i.e. EB formation) (Fig. 1). In addition, given that iPSC are potentially immortal, we felt that it would be important to be able to trace the identity of the cells as they pass through the manufacturing process and are distributed worldwide. While several methods are available, we used STR (single tandem based repeat) tracking, as CLIA certified laboratories perform this test routinely and results are rapidly available [46]. Examining our process, we determined that STR typing should be done on the donor sample prior to the start of the reprogramming step, and it should be matched to the final iPSC sample taken after the cryopreservation step at the end of the manufacturing process. Additional characterization was performed using quantitative PCR, which included examining residual plasmids clearance. Furthermore, sterility was carried out as a standard method on the starting materials (cord blood derived CD34+ cells) and the final manufactured cell product (see methods). Mycoplasma and endotoxin tests were also carried out as standard method on final iPSC products. Figure 2 illustrates the process flow diagram along with the in-process samples and associated tests. At the beginning of the process, it was important to test the sterility of CD34+ cells and purity of these cells using flow cytometry analysis (in-process QC1); it was also critical to test CD34+ cells for karyotype, take samples for STR analysis and matching with the final iPSCs, and evaluate the purity of CD34+ cells at the end of priming stage (in-process QC2). The karyotype analysis, in particular, was critical to ensure the starting materials undergoing the reprogramming process were normal. In-process QC3 was performed to evaluate the quality of iPSCs selected after the reprogramming based on the morphology of iPSCs [1] and an RT-PCR based plasmid clearance test. To confirm plasmid clearance, in-process QC4 was carried out at multiple passages before final expansion and banking. These multiple in-process tests were key to evaluate the quality of iPSCs before performing a comprehensive characterization of iPSCs through a wide range of QC tests [47, 48].

**Table 1:** Assays used to characterize the iPSC lines

Assay	Objective	Evaluation Criteria	Category	Tested iPSC Line
Assay Release				
Pluripotency Markers	Identity & Purity	SSEA-4 > 70 %, Tra-1-60 > 70 %, Tra-1-81 > 70 %, Oct3/4 > 70 %; Purity: CD34 < 5 %	Release assay	All lines
Karyotype Analysis	Safety	46, XX or 46, XY	Release assay	All Lines
Mycoplasma Testing	Safety	Negative	Release assay	All Lines
Sterility Testing	Safety	Negative	Release assay	All Lines
Endotoxin Testing	Safety	Standard QC release (<0.5 EU/ml)	Release assay	All lines
Vector Clearance	Safety	No trace of episomal plasmid DNA detected	Release assay	All lines
STR Genotyping	Purity & Identity	STR Profile of starting population and iPSC line are identical	Release assay	Al Lines
Cell Count & Viability	Viability	% viability >50; minimum cell number/vial	Release Assay	All Lines
Viral Panel Testing	Safety	Standard MCB Release Panel	Release Assay	LiPSC-GR1.1
Characterization Assays				
EB Formation	Identity & Potency	Detection of at least one marker per germ layer	FIO*	All lines
Gene Array Analysis	Identity	Clustering with established hPSCs	FIO*	All Lines
Colony morphology	Identity & Purity	Characteristic morphology of culture/colonies; lack of spontaneously differentiated cells	FIO*	All lines
Post-thaw Plating	Thawing efficiency and Viability	20+ colonies / vial (after 7 days or 50 % confluency)	FIO*	All Lines
HLA Typing	Identity	HLA-A, B, C, DRB1 and DQB1	FIO*	All lines
		Type		
CGH + SNP microarray	Identity	Amplifications and/ or deletions of specific genes	FIO*	LiPSC-GR1.1 and ER2.2
Whole Genome Sequencing	Identity	HiSeq X Human Whole Genome	FIO*	LiPSC-GR1.1 and ER2.2
		Sequence		

Table summarizes the tests that were performed on the three engineering run lines and the two cGMP lines (all). Note that the three engineering lines were generated at different times from the same donor sample (Female), while the two cGMP lines were generated from a different donor (Male)

• For Information Only (FIO)

An example of such a complete characterization is shown in Fig. 1 for one of the iPSC lines (LiPSC-ER2.1) produced during the engineering runs described before [1], and further detailed information on the tests conducted on the other lines is provided in Fig. 1 and Tables 1 and 2. Although such a comprehensive characterization may be deemed sufficient for these iPSC lines, we felt it was inadequate for the potential use of iPSC lines and that some additional important data needed to be collected. This included blood group (ABO and Rh) and HLA typing at high resolution. We noted that this was not part of the routine process of donor sample, and we were unable to return to the donor to obtain blood group data (Table 2) from one of the tissue acquisition sites.

**Table 2:** iPSC lines generated by Lonza: STR, HLA, and donor information

Line	M/F	STR	HLA Type	ABO/RH+/−	Ethnicity
LiPSC-GR1.1	M	Amelogenin (X, Y); vWA (16); D8S1179 (13, 14); TPOX (8); FGA (18, 25): D3S1358 (15); THO1 (6, 9.3); D21S11 (31.2, 33.2); D18S51 (14); Penta E (7, 11); D5S818 (11); D13S317 (9, 11); D7S820 (10, 11); D16S539 (11, 13); CSF1PO (12), Penta D (11, 13)	A*02/*03, B*07/*39, C*07/*15 DRB1*04/*08,DQB1*03/*04	O+	Hispanic
LiPSC-GR1.2					
LiPSC-ER2.1	F	Amelogenin (X); vWA (16); D8S1179 (14, 15); TPOX (8, 9); FGA (22, 23): D3S1358 (16, 18); THO1 (8, 9.3); D21S11 (24.2, 30.2); D18S51 (12); Penta E (10); D5S818 (11, 13); D13S317 (11); D7S820 (8, 10); D16S539 (11, 12); CSF1PO (10, 13), Penta D (9, 14)	A*03/*24, B*27/*27, C*01/*05 DRB1*01/*11,DQB1*03/*05	Inferred A	Caucasian
LiPSC-ER2.2					
LiPSC-ER2.3					

Table summarizes the information for identity and HLA typing and ethnic background that was collected. This data does not distinguish between clones from the same individual, and tracing and tracking of clones represents an issue that will require a solution to complement the barcoding and physical tracking methods we recommend

In summary, this minimal set of tests allowed us to track each line, define its basic characteristics and provide reasonable predictability as to the quality of the line and its potential to differentiate into desired differentiated cell types. The immunocytochemistry provided insight not only into the purity of the sample but also assessed the degree of contaminating cell populations and provided an unbiased method of comparing cells to established comparability criteria in the future.

However, while these tests may be necessary to evaluate a minimal level of the cells’ quality, we wondered if these would be sufficient to eliminate all unwanted cell types, and unfortunately this was unclear. Minor Karyotypic abnormalities or karyotypic abnormalities in a small percentage of the cells will be missed. Integration of genes used in the iPSC generation process will not be recognized, and changes or mutations in genes that are not functionally important at the pluripotent stage will be missed. We wondered if one could increase the predictability of the quality by adding additional tests, and more importantly, be prepared to add additional tests that may be required in the future. We have proposed three additional tests, including CGH array based on hybridization to complement the karyotyping; a transcriptome analysis; and a whole genome sequencing (WGS) assay that would complement the basic analysis and enable one to gather information as to critical parameters that need to be assessed for new lines.

### CGH + SNP Microarray Analysis

We selected a SNP hybridization based assay to provide a higher-resolution map of the state of the cells. We utilized both clones of the cGMP line and the three engineering lines generated from the same individual using the same cGMP compliant process to determine the utility of the methodology. We chose a CLIA-certified provider, and the results are provided in Table 3 (for GMP lines) and Table 4 (for ENG run lines). As can be seen in Table 3, although GR1.1 is karyotypically and phenotypically normal, it has several small duplications and deletions. (See Table 3 Panel A and B). Comparing the changes seen in GR1.1 and GR1.2, several of the duplications and deletions were common, suggesting that they preexisted prior to the initiation of the iPSC process. A smaller but significant number differed between the two samples and likely were generated during the process of cell line derivation or propagation in culture. A larger number of alterations were seen in the engineering clones, and what was surprising was the degree of non-overlap (i.e. uncommon aCGH) (Table 4). As with the ER clones, the detected changes were within the range described by other groups and included genes known to be affected in disease as well as genes known to be related to pathways important in disease [7, 8, 10]. Given the lack of overlap and because we had access to WGS and transcriptome analysis from cells at the same passage, we compared the results with those obtained by WGS (whole genome sequencing) and microarray expression. As discussed below, these changes did not appear to correlate with WGS data or alter transcription levels to any demonstrable extent. Although it is unclear as to the relevance of these changes, developing a database of the affected genes will allow us to determine if further changes occur as cells are propagated and whether changes in certain genes are common and related to the derivation process used, as has been suggested by some studies [7, 8, 10]. Indeed, in the relatively small sample size we noted that mutation in GNAS [49] were common in several lines.

**Table 3:** aCGH-SNP analysis of the iPSCs Manufactured during cGMP runs

Chromosome	Start	Stop	Genes	Cytoband	LiPSC GR-1.1	LiPSC GR-1.2
List A: Common changes observed in GR-1.1 and GR-1.2						
chr2	45,168,836	45,171,902	SIX3	P21	Present	Present
chr15	34,695,166	34,841,446	GOLGA8B, GOLGAA8A, MIR1233-1, MIR1233-2	q14	Present	Present
chr16	31,959,074	33,773,134	HERC2P4, LOC390705, TP53TG3, TP53TG3B, LOC653550, SLC6A10P	P11.2	Present	Present
chr20	57,463,534	57,464,754	GNAS	q13.32	Present	Present
chrX	130,813,232	131,201,564	LOC286467, MST4	q26.2	Present	Present
chrY	17,130,014	17,630,471		q11.221	Present	Present
Total No. of common aCGH						6
List B. Non overlapping changes observed GR-1.1 and GR-1.2						
chr2	81,631,218	84,380,876	LOC1720	p12 - p11.2		Present
chr2	166,180,491	166,815,270	SCN2A, CSRNP3, GALNT3, TTC21B	q24.3	Present	
chr3	46,620,840	46,622,617	TDGF1	p21.31	Present	
chr5	124,645,064	128,776,611	GRAMD3, ALDH7A1, PHAX, C5orf48, LMNB1, MARCH3, FLJ44606, MEGF10, PRRC1, CTXN3, FLJ33630, SLC12A2, FBN2, SLC27A6, ISOC1	q23.2 - q23.3		Present
chr6	47,311,409	54,276,380	CD2AP, GPR111, GPR115, OPN5, C6orf138, MUT, CENPQ, GLYATL3, C6orf141, RHAG, CRISP2, CRISP3, PGK2, CRISP1, DEFB133, DEFB114, DEFB113, DEFB110, DEFB112, TFAP2D, TFAP2B, PKHD1, MIR206, MIR133B, IL17A, IL17F, MCM3, PAQR8, EFHC1, TRAM2, LOC730101, TMEM14A, GSTA7P, GSTA2, GSTA1, GSTA5, GSTA3, GSTA4, ICK, FBXO9, GCM1, ELOVL5, GCLC, KLHL31, LRRC1, C6orf142, TINAG	p12.3 - p12.1		Present
chr7	155,596,206	155,601,974	SHH	q36.3		Present
chr10	114,549,196	117,896,129	VTI1A, LOC143188, TCF7L2, HABP2, NRAP, CASP7, C10orf81, DCLRE1A, NHLRC2, ADRB1, C10orf118, MIR2110, TDRD1, VWA2, AFAP1L2, ABLIM1, FAM160B1, TRUB1, ATRNL1, GFRA1	q25.2 - q25.3		Present
chr11	19,664,494	20,906,873	NAV2, LOC100126784, DBX1, HTATIP2, PRMT3, SLC6A5, NELL1	p15.1	Present	
chr12	19,001,106	19,925,941	PLEKHA5, AEBP2	p12.3	Present	
chr19	50,816,568	51,294,521	KCNC3, NAPSB, NAPSA, NR1H2, POLD1, SPIB, MYBPC2, FAM71E1, C19orf63, JOSD2, ASPDH, LRRC4B, SNAR-F, SYT3, LOC342918, SHANK1, CLEC11A, GPR32, ACPT	q13.33		Present
chrX	306,955	329,692	PPP2R3B	p22.33	Present	
Total No. of aCGH					5	6

aCGH-SNP analysis of the iPSCs GR1.1 and GR1.2 which are clones. Panel A identifies common changes detected and Panel B lists non- overlapping changes. The genes are color coded; Red- Known to be affected in disease. Black –Not reported as affected In database . Teal in a pathway related to diseaseNote the analysis was done at the same time on the same run. The clones were manufactured in two separate runs

**Table 4:** aCGH-SNP analysis of the iPSCs Manufactured during the Engineering Runs. aCGH-SNP analysis of the iPSCs ER2.1, ER2.2, ER2.3 which are clones. Panel A identifies common changes detected and Panel B lists non- overlapping changes. The genes are color coded; Red- Known to be affected in disease. Black –Not reported as affected In database . Teal in a pathway related to disease. Note the analysis was done at the same time for ER 2.2 and ER 2.3 and on a separate run for ER2.1. on the same run. The clones were manufactured in three separate runs

Chromosome	Start	Stop	Overlap	Genes	Cytoband	LiPSC ER-2.1	LiPSC ER-2.2	LiPSC ER-2.3
A. Common aCGH between LiPSC ER-2.1, LiPSC ER-2.2, and LiPSC ER-2.3 lines								
chr16	34,482,042	34,743,643	100	LOC283914, LOC146481, LOC100130700	p11.2 - p11.1	Present	Present	Present
Total No. of Common aCGH						1		
B. Uncommon aCGH between LiPSC ER-2.1, LiPSC ER-2.2, and LiPSC ER-2.3 lines								
chr2	192,581,125	199,052,600	SDPR, TMEFF2, PCGEM1, SLC39A10, DNAH7, STK17B, HECW2, CCDC150, LOC100130452, GTF3C3, C2orf66, PGAP1, ANKRD44, SF3B1, COQ10B, HSPD1, HSPE1, HSPE1-MOBKL3, MOBKL3, RFTN2, MARS2, BOLL, PLCL1		q32.3 - q33.1	Present		
chr2	193,974,618	199,390,001	SLC39A10, DNAH7, STK17B, HECW2, CCDC150, LOC100130452, GTF3C3, C2orf66, PGAP1, ANKRD44, SF3B1, COQ10B, HSPD1, HSPE1, HSPE1-MOBKL3, MOBKL3, RFTN2, MARS2, BOLL, PLCL1		q32.3 - q33.1			Present
chr4	45,882	68,211	ZNF595, ZNF718		p16.3		Present	
chr4	62,069,285	63,216,102	LPHN3		q13.1		Present	
chr5	145,468,046	147,372,489	PLAC8L1, LARS, RBM27, POU4F3, TCERG1, GPR151, PPP2R2B, STK32A, DPYSL3, JAKMIP2, SPINK1, SCGB3A2, C5orf46		q32			Present
chr5	146,215,672	147,372,489	PPP2R2B, STK32A, DPYSL3, JAKMIP2, SPINK1, SCGB3A2, C5orf46		q32		Present	
chr5	156,407,838	156,619,217	HAVCR1, HAVCR2, MED7, FAM71B, ITK		q33.3			Present
chr6	296,674	418,057	DUSP22, IRF4		p25.3	Present		
chr6	296,674	462,491	DUSP22, IRF4		p25.3			Present
chr7	82,012,870	85,939,933	CACNA2D1, PCLO, SEMA3E, SEMA3A, SEMA3D		q21.11			Present
chr7	121,256,234	122,561,644	PTPRZ1, AASS, FEZF1, LOC154860, CADPS2, RNF133, RNF148		q31.32		Present	
chr8	7,220,322	7,752,586	ZNF705G, DEFB4B, DEFB103B, DEFB103A, SPAG11B, DEFB104B, DEFB104A, DEFB106B, DEFB106A, DEFB105B, DEFB105A, DEFB107A, DEFB107B, FAM90A7, FAM90A14, FAM90A13, FAM90A19, FAM90A18, FAM90A8, FAM90A9,FAM90A10, SPAG11A, DEFB4A		p23.1		Present	
chr9	129,453,130	129,725,863	LMX1B, ZBTB43, ZBTB34, RALGPS1		q33.3		Present	
chr9	138,157,660	138,283,617			q34.3			Present
chr11	2,016,471	2,024,343	H19, MIR675		p15.5			Present
chr11	26,859,414	28,019,072	FIBIN, BBOX1, CCDC34, LGR4, LIN7C, BDNF-AS1, BDNF		p14.2 - p14.1			Present
chr11	67,374,414	67,378,114	NDUFV1		q13.2		Present	
chr12	19,039,306	20,040,127	PLEKHA5, AEBP2		p12.3 - p12.2		Present	
chr13	54,124,303	58,474,271	MIR1297, PRR20B, PRR20C, PRR20D, PRR20E, PRR20A, PCDH17		q14.3 - q21.1		Present	
chr14	107,145,681	107,182,658			q32.33	Present		
chr15	22,587,129	22,849,189	GOLGA8DP, GOLGA6L1, TUBGCP5		q11.2		Present	
chr20	57,463,534	57,464,754	GNAS		q13.32		Present	Present
chr22	19,749,830	19,758,306	TBX1		q11.21		Present	
chr22	51,063,432	51,110,017	ARSA		q13.33		Present	
chrX	306,955	319,352	PPP2R3B		p22.33	Present		
chrX	443,311	445,970			p22.33		Present	
chrX	8,514,158	8,547,642	KAL1		p22.31		Present	
chrX	53,449,448	53,459,515	SMC1A, RIBC1, HSD17B10		p11.22			Present
Total No. of aCGH						4	15	10

### Microarray Analysis

We reported a whole genome expression analysis of iPSC lines generated during process development, training runs, engineering runs and GMP manufacturing runs [1, 14]. Here we compared gene expression of 10 iPSC lines generated from the same manufacturing process. Samples of the cells were collected for RNA extraction and whole genome expression analysis conducted using Illumina Bead Array platform (Human HT-12 v4 Expression BeadChip). We have previously shown that this platform is suitable for reliable and robust detection of differential gene expression in a large number of samples [50, 51]. As a control, we included additional iPSC and ESC lines as well as a CD34+ sample, from which one iPSC line was derived and the NSC differentiated from the iPSC. The list of samples analyzed is reported in Supplemental Table 1, and the entire gene expression profile is reported in Supplemental Table 2, available upon request. Initial data processing was done in GenomeStudio software as previously described [52]. Whole genome expression raw data, normalized and non-normalized data can be accessed through Gene expression accession number of GSE72078. The quality control tests and the various analyses we performed are summarized in Table 5.

**Table 5:** Microarray Analysis for pluripotent cells

Data Set analysis
QC Tests
Total reads
Intensity range
Distribution
No detected > 100 RFU
Normalization
Other Results
Overall profile (relatedness)
Chromosomal Bias
M/F
X chr inactivation
Pluritest
Pluripotent marker set
Contaminating cell detection
Incomplete programming
Gene Sets
MicroRNA
Mitochondrial profiling
Pathway analysis
Positional markers
Imprinted genes

The table summarizes the routine QC tests and the results with GR1.1. Note that both gene subsets as well as examination of overall quality can be inferred using tests such as Pluritest, correlation co-efficients and unbiased hierarchical clustering. The data quality is improved by comparing to a database and with sufficient numbers of lines one can establish ranges for a comparability assay

**Fig. 3:**
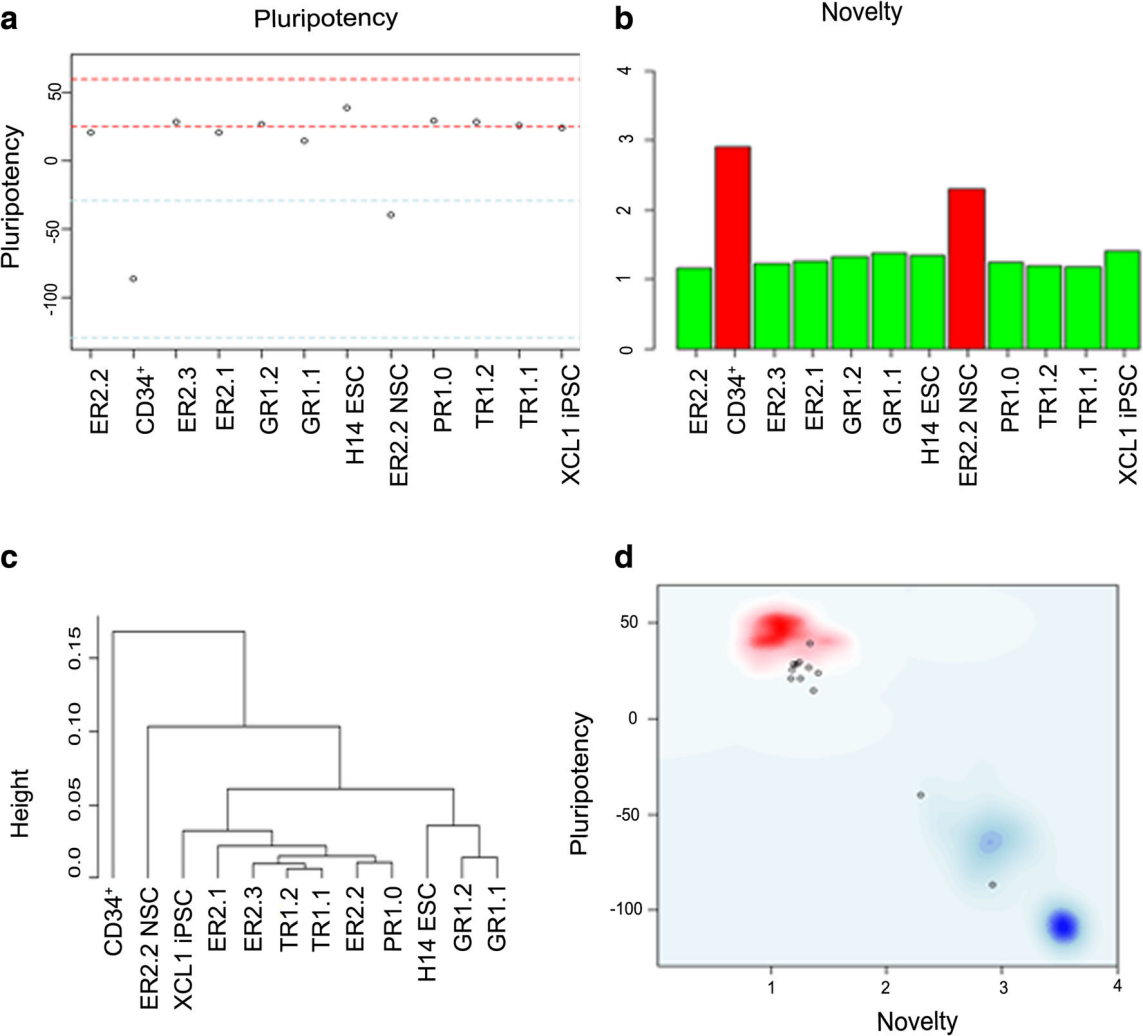
Pluritest analysis of test and control samples. **a**. Model-based multi-class pluripotency score: pluripotency score between *red lines* indicates a 95 % pluripotency signature. Samples between *blue line* indicates 95 % of non-pluripotent samples. Line PR1.0, TR1.1, TR1.2, GR1.1, GR1.2, ER2.1, ER2.2, ER2.3, XCL1iPSC and H14 ESC are all localized between or close to the red lines, hence all pluripotent. The CD34^+^ cord blood line and ER2.2 NSC are (negative controls) are located between the blue lines demonstrates non-pluripotent signature. **b**. Novelty Score: This score is based on well-characterized pluripotent samples, color-coded green and non-pluripotent samples *color-coded red*. All iPSC and ES samples are in *green* demonstrates pluripotent samples, whereas CD34^+^ *cord blood line* and ER2.2 NSC demonstrates non-pluripotent *color-code red*. **c**. Hierarchical Clustering of vst-transformed samples: Samples were transformed using variance stabilizing transformation (VST) and robust spline normalization _ENREF_35[40]. Distance on x -axis is based on Pearson correlations. **d**. Overview: This combines novelty score on X-axis and Pluripotency score on y-axis. The *red background*, where the iPSC and ESC samples are located, suggest the empirical distribution of pluripotent cells. CD34^+^ *cord blood line* and ER2.2 NSC are located closer to the non-pluripotent background *colored blue*

First, we performed the quality control of our data set. The average number of detected genes for all samples was highly similar: 11,798.4 ± 701.6 (detection *p*-value < 0.01; mean ± standard deviation; non-normalized data) and 14,661 ± 710.1 (detection *p*-value < 0.05; mean ± standard deviation; non-normalized data), and no wide discrepancies in hybridization signal intensity distributions were observed (not shown). The quality of the iPSC was verified by submitting the samples for the Pluritest assay and the results are shown in Fig. 3. Pluritest verification of iPSC line PR1.0, TR1.1, TR1.2, GR1.1, GR1.2, ER2.1, ER2.2, ER2.3 and XCL1iPSC samples fall within the same pluripotency range (red lines) of ESC samples and iPSC samples used to develop Pluritest, as does our added positive controls H14 ESC. The CD34^++^ cord blood line and ER2.2 NSC fall into non-pluripotent range (blue lines), demonstrating that our iPSC lines correlate with known iPSC and ESC (Fig. 3a).

In order to visualize the overall strength of measured signal across samples and identify presence of potential outliers, we plotted the signal-to-noise ratios and high-end intensity variation (95th percentile of signal intensity, P95) (Fig. 3b) in non-normalized data. Signal intensity was similar in all tested samples and no outliers were detected, suggesting matching quality across microarray samples. Based on technical and biological variation, all our iPSC and ESC samples falls within the novelty score (green), while CD34^++^ cord blood cells and the ER2.2 NSC fall outside the novelty score (red) (Fig. 3b). Next, we calculated the pairwise correlation coefficients (*r*^2^) to determine the overall relatedness of samples (data now shown). The correlation coefficients between the iPSC samples manufactured using the cGMP compliant process was greater than 0.9 [1], and no significant difference in gene expression profiles at a global level was observed among these iPSC lines and the ESC or iPSC lines previously generated using similar or different reprogramming methodologies [1, 50]. We then performed unsupervised one-way hierarchical clustering analysis to group averaged samples according to the degree of gene expression similarity (Fig. 3c). The results displayed three distribution features: starting material (CD34+ cells used to generate iPSC), pluripotent stem cells (iPSC and ESC), and differentiated neuronal cells. Together, these findings indicated good overall quality of the microarray data. Combination of novelty score and pluripotency score (Fig. 3d) illustrates iPSC and ESC samples are grouped together, suggesting an empirical distribution of pluripotent cells (red background). CD34^++^ cord blood cells and ER2.2 NSC are located closer to the non-pluripotent (blue background) (Fig. 3d). These results also confirm that Pluritest can be used as another tool to verify pluripotency of newly derived iPSC.

In addition to utilizing Pluritest, we reasoned we could also identify subsets of genes which have been identified as being PSC cell-specific and compare their levels of expression with that in other PSC that have been well characterized, such as XL1 [50, 51]. In addition, markers of CD34+ cells as well as markers of trophoblast and early differentiating cells can be examined (see supplementary materials) to assess completed transformation and the absence of contaminating cells. We note that the Illumina chips include microRNA and our isolation process preserves small RNA species, allowing us to use the same array system to assess microRNA expression profiles of the cells. Comparison between the two lines and the other lines included in this analysis showed that the lines were similar in their expression profiles and did not show the presence of differentiated cell markers (data not shown). In particular, the expression of positional markers (such as HOX genes) was absent.

Another factor that may alter the behavior of PSC, which is unlikely to be assessed by routine tests, is the expression of imprinted genes. We therefore extracted the list of published imprinted genes and compared the expression of these genes in the entire sample set (Supplemental Table 3, available upon request). Overall the expression patterns were similar, though we noted a significant difference in the expression of NNAT in XCL1 iPSC and GR1.2. While the relevance of this observation is unknown, we believe following the levels of these genes may be important given their association with disease.

Evaluation of gene expression based on a panel of approximately 325 markers (Supplemental Table 3) -- including markers of pluripotency, gender, imprint, endoderm, mesoderm and ectoderm) revealed (1) no difference between the male or female lines, (2) no change in the expression of imprinted genes, and (3) highly expressed level of several pluripotency markers including *Oct4*, *Nanog* and *Sox2* in all iPSC samples (1). Overall, gene expression profiling of the lines generated using cGMP compliant process was similar and similar to previously reported iPSC and ESC lines [52].

To further analyze gene expression data, we compared expression of genes associated with functional pathways. As an example we compared the levels of genes involved in Cell Cycle, as well as genes coded for Transcription factors (TF) and Growth Factor Receptors (GFR) between iPSC line ER2.2 and GR1.1. Our results (Supplemental Table 4) showed no significant differences in gene expression in these pathways between the 2 iPSC lines. (Supplemental Table 4).

We also reasoned that one could identify the sex of the individual using expression of Y chromosome genes, and assess if X chromosome inactivation had occurred in female samples by examining expression of XIST and the levels of expression of X chromosome genes and comparing them with cell lines that have demonstrated X chromosome inactivation or activation. While this is by no means a definitive test, it allows one to highlight where further testing is required. Further information on imprinting can be obtained by identifying the differences between sequences between the two alleles of the imprinted genes by WGS and determining whether expression was from the maternal or paternal allele. Although we did not perform such an analysis in this work, such analysis can be readily performed if considered necessary.

An additional analysis that could readily be performed was assessing if the deletions and duplications reported in the WGS and SNP/CGH array tests had an effect on gene expression by preparing a list of deletions and duplications in GR1.1 and GR1.2 and examining the expression of those genes that are known to be expressed at the PSC stage. No significant change in expression was detected (Sup Table 3).

We have previously shown that rather than comparing individual gene expression one can examine the overall expression of a particular signaling pathway or a subset of genes related to a particular disease and examine if there are any obvious differences in expression [50, 51]. For iPSC, we reasoned that expression of mitochondrial-related genes and genes related to cell death may be sensitive predictors of growth rate and proliferation as data on lines is collected. Furthermore, mitochondrial mutations account for a large group of hereditary disorders. We therefore examined the subset of mitochondrial genes to examine if any significant changes were observed when compared to other iPCS and ESC lines. These analyses revealed no significant change in the expression of mitochondrial-related genes (data not shown).

Overall our results suggest that microarray analysis is a relatively inexpensive method that allows one to rapidly evaluate pluripotency and presence of contaminating cells using a variety of methods. One can compare expression of specific subsets of genes that may be critical for the use of these cells for a specific purpose and importantly provide a referral database to compare the ongoing evolution of the cells as they are maintained in culture. Collection of such a database may allow us to develop comparability criteria and acceptance and release assay cut-offs for cell processing.

### Whole Genome Analysis

**Fig. 4:**
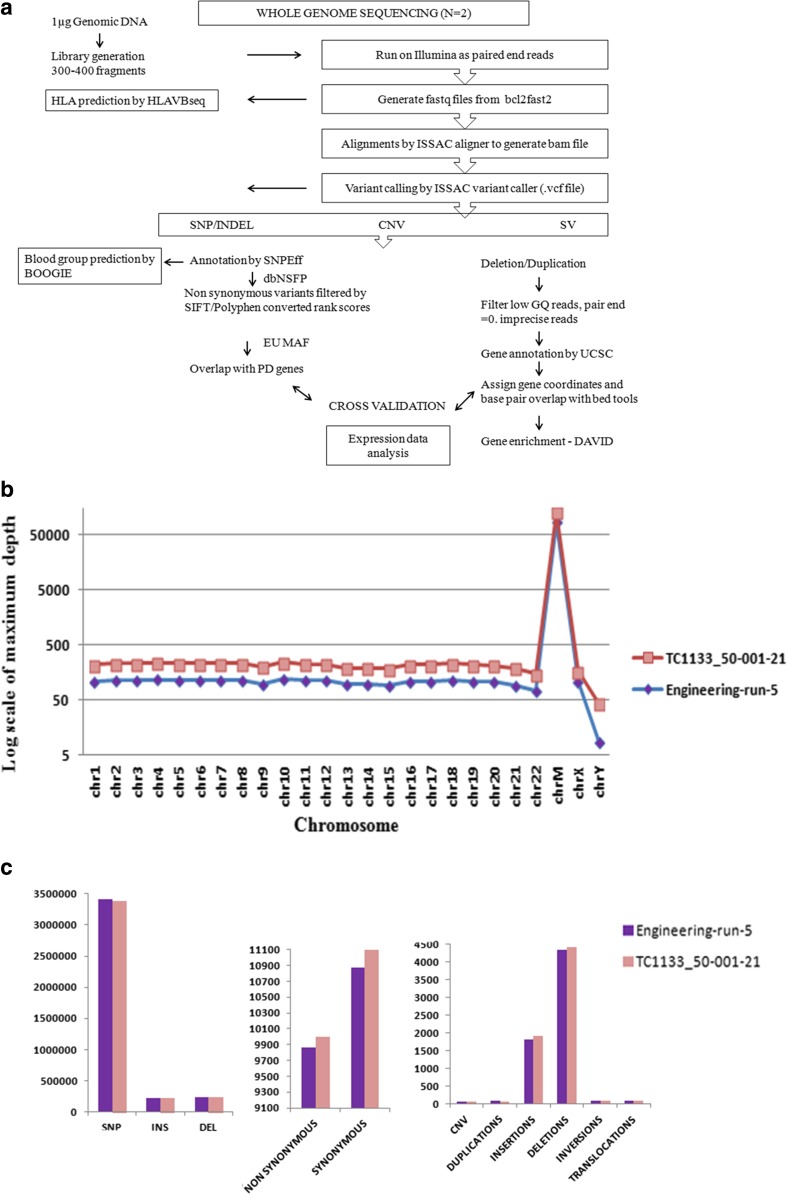
Whole Genome analysis conducted on two iPSC lines generated using the cGMP compliant manufacturing process. **a**. WGS data characterisation pipeline. This figure outlines the work flow followed in this study. The filters applied at various stages have been mentioned in the methods section. The fastq files were aligned using Isaac aligner to generate bam file. The bam file generated was checked for its mapping quality using samstat. The fastq files were also used for prediction of HLA types using HLAVBseq. The variants called by Issac variant caller was annotated using SnpEff and this data was used for predicting blood groups using BOOGIE. Only non-synonymous variants were considered for its implication with PD and cross validated with expression data. Structural variation (deletions and duplications) were filtered (see methods) and annotated for genes using UCSC. This data was cross validated with expression and microarray data. The differentially expressed genes were verified for gene enrichment relevant to disease and pathway through DAVID. **b**. The x-axis shows the various chromosomes and Y-axis represents the max-depth computed by Issac Variant Caller across each chromosome on a log scale for both the cell lines under study. **c**. Bar graph representing number of variants identified for SNPs, small Insertions, small deletions, synonymous, non-synonymous variants, CNVs and different types of structural variants including duplications, large insertions (length > 50), large deletions (length > −50), inversions and translocations. Deletions were higher in number than other types of SVs

We prepared genomic DNA and used it to perform the WGS on two iPSC lines, one male and one female, to assess their overall status. The data was analyzed using the pipeline summarized in Fig. 4a. A list of tests that we have performed using these data sets is summarized in Table 6. Table 7 summarizes the QC parameters evaluated. Both the lines passed the standard QC parameters.

**Table 6:** Whole Genome Analysis for pluripotent cells

Data Set analysis
QC Tests
Total reads
Unmapped reads
Mapped reads
Chr. coverage
Error Rate
Other Results
Mutations detected
Mitochondrial Seq
Comparison with CGH
STR verification
Distinguishing alleles
HLA
Minor Blood groups
Other transplant antigens
Other gene subsets
Plasmid/Viral Insertion sites
Utilizing unmapped data
Mycoplasma
Other infectious agents
Foreign DNA

The table summarizes the routine QC tests and the results with GR1.1. Note that both gene subsets as well as examination of overall quality can be inferred using tests such as Pluritest, correlation co-efficients and unbiased hierarchical clustering. The data quality is improved by comparing to a database and with sufficient numbers of lines one can establish ranges for a comparability assay

**Table 7:** Basic QC analysis of WGS data. Basic QC and mapping quality summary statistics for both the cell lines computed using samtools and Samstat

QC anlysis Summary	LiPSC-ER2.2	LiPSC-GR1.1
Total reads	840146878	898394686
Total reads mapped	33656253	768473456
Mean depth	34.49	30.83
deduplicated reads	90375272	157504614
Total reads after removing duplicates (%)	749771606 (89.2 %)	740890072 (82.5 %)
Reads mapped to human genome	693727158	613291081
MAPQ > =30	86.9 %	79.6 %
MAPQ <30	0.1 %	0.0 %
MAPQ <20	0.5 %	0.5 %
MAPQ <10	0.7 %	0.7 %
MAPQ <3	0.3 %	0.3 %

The average number variants identified in both cell lines were similar and showed uniform patterns in the number of SNP, INS, DEL, synonymous and non-synonymous variants. As the number of deletions was higher than insertions or translocations, these were analyzed further (Fig. 4c).

The maximum depth of coverage obtained across each chromosome for both cell lines are shown in Fig. 4b. Higher depth of coverage in mtDNA was observed in both cell lines, as is to be expected. Y chromosome in LiPSC-ER2.2 dipped as expected in the female line. The total of 36 variants of Y chromosome overlapped with the X chromosome from the LiPSC-GR1.1 (male line). This highlights the issues with assignment of sequence data.

**Fig. 5:**
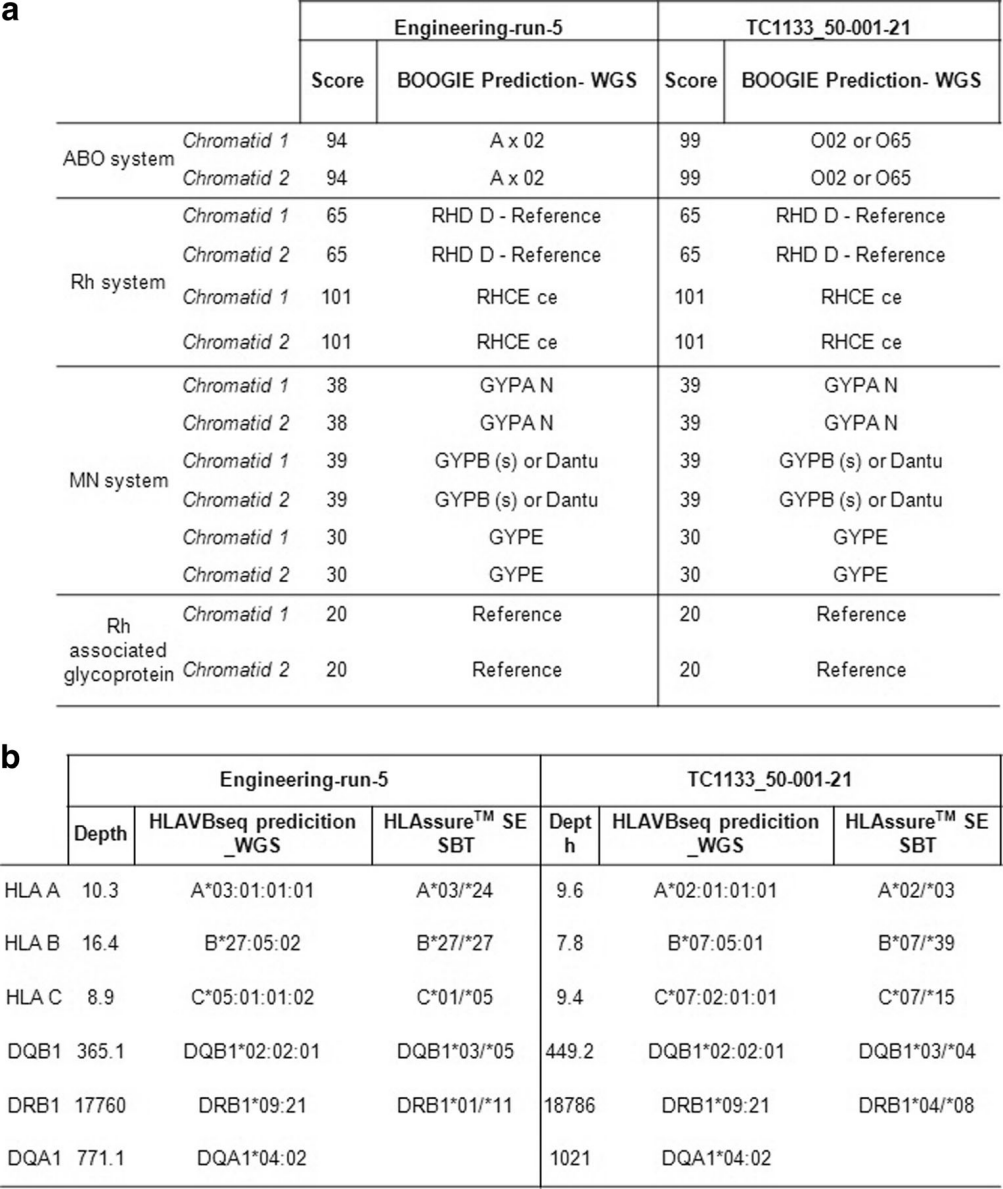
Extracting blood type and HLA from the whole genome sequencing data. **a**. Table showing the blood group predicted by BOOGIE for four different blood group systems namely ABO, Rh, MN and Rh associated glycoprotein with the score for both the cell lines. The validity of this approach is provided as means of comparison with the actual available donor information. **b**. Table showing HLA types predicted in-silico by HLAVBseq using WGS data for both the cell lines along with their depth. The predicted HLA type obtained using the WGS data is compared with the results obtained using the HLA AssureTM SE SBT kit

Extracting Blood type from the whole genome sequencing data was predicted by using BOOGIE for four different blood group systems, namely ABO, MNS, Rh and RhAG (Fig. 5a). The two cell lines LiPSC-ER2.2 and LiPSC-GR1.1 were distinguished based on their ABO system, showing blood group A and O respectively with a high confidence score of 94 and 99. The blood group of LiPSC-GR1.1 matched with that of the donor data LiPSC-GR1.1 for ABO system (blood group O), whereas donor information for LiPSC-ER2.2 was unavailable for comparison.

Comparing predictions across platforms, we note that SNP Chip analysis would have predicted the phenotype as O. This is because the SNP Chip does not pick up the insertions while WGS does, indicating the power and sensitivity of the WGS. The same program or similar program can be used to extract information for the thirty minor blood group antigens that have been described [53].

As with extracting Blood groups, we reasoned that it should be possible to use the data to identify the HLA phenotypes of the cell lines, and we used HLAVBSeq program (Fig. 5b.). The predicted HLA types had a resolution up to 8 digits. Both iPSC cell lines could be distinguished based on their class I HLA types along with their average depth of coverage, whereas class II HLA types show similar genotype but different depths.

The HLA prediction using the WGS and HLAssure^TM^ SE SBT platforms was comparable for both the lines with two differences in DQB1 and DRB1 loci . The genotypes for these two loci in LiPSC-ER2.2 are DRB1*01/*11 and DQB1*02:02:01 (365.1): DQB1*03/*05.

Likewise for LiPSC-GR1.1, DQB1*02:02:01 (449.2): DRB1*04/*08, DRB1*09:21 (18785.9): DQB1*03/*04. From these results it can be inferred that the WGS gives higher resolution of the HLA prediction, and this with the coverage values provide confidence in call. The WGS allows us to identify the cis or trans type of the HLA types, which is not feasible by the standard methods of assessment. The DQA1-DQB1 haplotype in both cell lines was found to be in trans-isoform.

Our interest was studying Parkinson’s disease (PD), and we wanted to see if a focused analysis of the data could be performed. So we prepared a list of PD-related genes to assess their status as an example to how a report can be prepared for specific, disease-related gene sets.

Both iPSC lines showed 23 unique genes relating to PD, after filtering for damaging effects of the amino acid substitution, predicted in-silico by SIFT and Polyphen converted rank scores computed by dbNSFP. These filtered variants were verified with the expression data. LiPSC-ER2.2 genes *SYNJ1* and *E1F4G1* have been implicated in PD when compared to MIM disease database. Whereas line LiPSC-GR1.1 showed two variants for *MAPT* gene (rs63750417) and one variant with *SYNJ1* gene (rs2254562), which showed its association with PD (Table 8).

**Table 8:** Identification of PD implicated genes from WGS. The table shows the genes that are identified by WGS, having damaging SIFT and polyphen scores that are implicated in PD. These genes were cross verified for the changes in their RNA expression

SAMPLE	SYMBOL	CHR	POS	SNP ID	SIFT	POLYPHEN 2	1000Gp3_EUR_AF	ER1.2 (F)	GR1.1(M)	H9(F)	H7(F)	NCRM6(F)	MIM_disease
LiPSC-ER2.2	SYNJ1	21	34059352	rs2254562	0.43	0.629	0.29	56		69	65	61	[MIM:615530]Parkinson disease
	EIF4G1	3	184039151	rs367754765	0.912	0.647		1483		1740	1426	1404	[MIM:614251]Parkinson disease
LiPSC-GR1.1	MAPT	17	44060775	rs63750417	0.91	0.72	0.24		4	37	26	10	[MIM:600274]Parkinson Disease
	SYNJ1	21	34059352	rs2254562	0.43	0.63	0.29		84	69	65	61	[MIM:615530]Parkinson disease
	MAPT	17	44061278	rs17651549	0.91	0.72	0.24		4	37	26	10	[MIM:260540]Parkinson-dementia syndrome

* These variants did not show its signature at Clinvar database

The structural variants identified in WGS were cross-validated with the results obtained from other platforms such as microarray and expression data. LiPSC-ER2.2 showed no overlap of the data obtained from WGS in comparison to SNP data in both unfiltered and filtered data (only one clone of LiPSC-ER2.2 showed overlap of *PPP2R2B* gene across both these platforms). The LiPSC-GR1.1 showed overlap of *CSRNP3* and *PPP2R3B* in the unfiltered data from WGS. But the genes were removed due to bad quality in the filtered set of the WGS. The *CSRNP3* gene had a discrepancy that was considered as GAIN in microarray data, and WGS data shows it to be a deletion event. This data is summarized in Table 9 along with the log fold change in relation to the control lines H9_H7 and NCRM6.

**Table 9:** Cross-validation of the structural variants identified across various platforms: WGS, Microarray, and SNP analyses

	Microarry platform	EVENT	ovelap with WGS-unfiltered	overlap with expression data	H9_H7	NCRM6
LiPSC-ER2.2	IRF4	DEL	no	yes	0.27	0
	DUSP22	DEL	no	yes	0.64	0
	LOC283914	AMP	no	yes	-	−1
	LOC146481	AMP	no	yes	−4	0
	LOC10013070	AMP	no	yes	2.85	-
	PPP2R3B	AMP	no	yes	1.3	0
LiPSC-GR1.1	SIX3	GAIN		yes	1	4
	SCN2A	GAIN		yes	2	-
	GALNT3	GAIN		yes	0	3
	TTC21B	GAIN		yes	−13	1
	CSRNP3	GAIN	yes (but as del)	yes	0	3
	TDGF1	GAIN		yes	0	−1
	NAV2	GAIN		yes	0	1
	HTATIP2	GAIN		yes	0	0
	SLC6A5	GAIN		yes	-	-
	NELL1	GAIN		yes	1	0
	LOC100126784	GAIN		yes	1	0
	DBX1	GAIN		yes	−1	-
	PRMT3	GAIN		yes	0	0
	PLEKHA5	GAIN		yes	0	0
	AEB2	GAIN				
	GOLGA8B	LOSS		yes	−2	0
	GOLGA8A	LOSS		yes	-	-
	MIR1233-1	LOSS				
	MIR1233-2	LOSS				
	HERC2P4	LOSS		yes	1	2
	LOC390705	LOSS		yes	0	1
	TP53TG3	LOSS		yes	0	2
	TP53TG3B	LOSS				
	LOC653550	LOSS		yes	-	−1
	SLC6A10P	LOSS		yes	−1	−1
	GNAS	GAIN		yes	0	0
	PPP2R3B	GAIN	yes	yes	0	1
	LOC286767	GAIN				
	MST4	GAIN		yes	−1	0

To verify if the genes overlapping with structural variants identified from WGS- filtered data set showed any implication with known diseases or pathways, we tested for gene enrichment through DAVID. The line LiPSC-ER2.2 showed no signals with disease association or pathways for deletion event. For duplication event, *HTR6*, *GRIK3*, *OPRD1* were found to be associated with chemdependency and was enriched for pathway Eicosanoid Metabolism but with only 2 genes (*CYP2J2*, *PTGER3*).

The LiPSC-GR1.1 line showed no disease-specific enrichment but was enriched for T Cytotoxic Cell Surface Molecules, T Helper Cell Surface Molecules pathways for deletion event and no enrichment was found with the duplication event (Table 10).

**Table 10:** Structural variants identified by WGS and its enrichment in any disease related pathways (if any)

	Event	No. after filter	Max size	Min size	no ofgenes log2 ratio >1.5 with H7_H9	no of genes log2 ratio >1.5 with NCRM6	overlap if any*	DAVID-OMIM disease GE	DAVID- pathway GE
LiPSC-ER2.2	DEL	653	16797153	89	11	18	no	no	no
	DUP	58	387	128375322	28	53	6	Chemdependency (Genetic_Association_DB_Disease_Class): HTR6, GRIK3, OPRD1	Eicosanoid Metabolism (BIOCARTA)-CYP2J2,PTGER3
LiPSC-GR1.1	DEL	555	16797153	122	24	20	4	no	T Cytotoxic Cell Surface Molecules, T Helper Cell Surface Molecules(BIOCARTA)-CD3D, THY1
	DUP	58	17376	462	0	0	no	NA	NA

To check the status of the imprinted genes, the list of published imprinted genes were obtained from the database (http://www.geneimprint.com). Genes with the status of imprinted and predicted were considered for the analysis. In LiPSC-ER2.2 cell line, a total of 148 of 203 genes were identified with an adequate depth of coverage. Similarly for LiPSC-GR1.1 cell line, a total of 147 of 203 genes were identified.

We also examined the expression of these imprinted genes at the iPSC stage by microarray analysis. LiPSC-ER2.2 and LiPSC-GR1.1 exhibited detectable expression of 121 and 120 genes respectively (Table 11). The number of homozygous, heterozygous SNPs, INDELS for these genes and its documented inheritance are given in Table 11. Thus, the variants that show the differences in both the chromosomes for imprinted genes could be extracted. Given the parental information we would be able to phase the data to identify the paternal or maternal specific allele expression in future by combining this data with RNA sequencing data.

**Table 11:** Summary of imprinted genes identified by WGS

Imprinted genes	Status	Expressed allele	LiPSC-ER2.2				LiPSC-GR1.1			
			Allelic depth	SNP-HET	INS-HET	DEL-HET	Allelic depth	SNP-HET	INS-HET	DEL-HET
ABCC9	Predicted	Maternal	27.4	50	2	3	26.0	54	5	5
ABCG8	Predicted	Maternal	27.0	73	4	5	25.8	65	3	3
ACD	Predicted	Maternal	26.5	2			23.0	1		
ADAMTS16	Predicted	Maternal	28.0	254	20	23	26.0	319	20	24
AIM1	Imprinted	Paternal	29.3	82	4	10	23.4	54	4	
ALDH1L1	Predicted	Maternal	25.5	148	3	9	25.9	47	6	6
ANO1	Imprinted	Maternal	22.8	42	1	2	26.0	1		
APBA1	Predicted	Paternal	29.0	118	8	11	27.5	8		4
ATP10A	Imprinted	Maternal	25.3	256	12	18	24.0	203	10	20
B4GALNT4	Predicted	Maternal	24.3	17	2	1	23.4	21	3	1
BLCAP	Imprinted	Isoform Dependent	23.8	12	5		26.5	4	3	1
BMP8B	Predicted	Paternal	23.4	3			22.4	10	2	
BTNL2	Predicted	Maternal	27.8	34	2	3	25.5	80	4	6
C10orf91	Predicted	Maternal	25.5	1			35.0			
C20orf20	Predicted	Maternal	25.4	14	1	2	27.8	14	1	2
C9orf116	Predicted	Paternal					18.9			
C9orf85	Predicted	Paternal	27.9	3			26.8			
CCDC85A	Predicted	Paternal	27.1	245	19	14				
CDH18	Predicted	Paternal	28.3	893	83	94	25.6	244	28	29
CDK4	Predicted	Maternal	24.3	1		2	21.8	3		1
CDKN1C	Imprinted	Maternal	27.0	1			15.0			
CHMP2A	Predicted	Maternal	31.0	1			23.5	4		
CHST8	Predicted	Maternal	24.3	96	4	6	25.1	121	8	8
COL9A3	Predicted	Maternal	23.1	50	2	2	21.9	34	6	
CPA4	Imprinted	Maternal	25.9	2			22.5	8	1	1
CSF2	Predicted	Maternal	19.0	1			27.0			
CYP1B1	Predicted	Paternal	26.1		1		28.4	9	2	
DDC	Imprinted	Isoform Dependent	26.3	190	11	9	25.6	138	5	8
DGCR6	Imprinted	Random	23.2	2			24.1	8		1
DGCR6L	Imprinted	Random	22.6				22.1	6		1
DIRAS3	Imprinted	Paternal					15.0	1		
DLGAP2	Imprinted	Paternal	25.7	4		2	24.8	21	2	1
DLK1	Imprinted	Paternal	20.4	9	1		21.7			
DLX5	Imprinted	Maternal	25.3	1	1	1	23.7	1		1
DNMT1	Imprinted	Paternal	23.2	36	1	9	23.0	50	3	5
DVL1	Predicted	Maternal	26.3	1		1	23.6	3		1
E2F7	Predicted	Maternal	30.2	28			29.6	23		1
EGFL7	Predicted	Paternal	21.2	1			22.1	23		
EVX1	Predicted	Paternal	24.0							
FAM50B	Imprinted	Paternal	31.0	1			25.0	1		
FBRSL1	Predicted	Maternal	23.7	128	7	8	20.5	10		3
FERMT2	Predicted	Paternal	29.0	6			28.2	86	11	3
FGFRL1	Predicted	Maternal	21.6	19	1	2	22.9	17	4	2
FOXF1	Predicted	Maternal	18.6	2	1	1	22.5			1
FOXG1	Predicted	Paternal	27.8	54	4		25.0			
FUCA1	Predicted	Paternal	27.7	1						
GAREM	Predicted	Paternal	27.9	36	4	3				
GATA3	Predicted	Paternal	24.3	28	4	1	21.0	9	1	
GDAP1L1	Imprinted	Paternal	27.0	3		1	24.3	2		1
GFI1	Predicted	Paternal	26.0	9	2		23.4	10	3	
GLI3	Predicted	Maternal	27.8	234	19	16	25.5	299	21	18
GLIS3	Imprinted	Paternal	26.3	517	22	36	25.5	611	25	37
GNAS	Imprinted	Isoform Dependent	28.0	68	8	4	26.8	32	2	4
GPT	Predicted	Maternal	15.0	1			20.5			
GRB10	Imprinted	Isoform Dependent	27.1	246	13	20	24.9	180	10	9
H19	Imprinted	Maternal	21.5	6			22.2	6		
HOXA11	Predicted	Maternal	23.1	4		1	24.8	1		
HOXA2	Predicted	Maternal	31.0				19.5	1		
HOXA3	Predicted	Maternal	26.5	5	3	1	22.7	11	2	1
HOXA4	Predicted	Maternal	21.0				25.0			
HOXA5	Predicted	Maternal	19.0				37.0		2	
HOXB2	Predicted	Maternal	25.2	3	1	1	27.5			
HOXB3	Predicted	Maternal	25.2	15	1	2	22.9	7		
HOXC4	Predicted	Maternal	26.3	13	6	5	24.1	24	1	8
HOXC9	Predicted	Maternal	23.3	4			21.0	1		
HSPA6	Predicted	Maternal	23.5	1			23.8	4		
HYMAI	Imprinted	Paternal	27.0				21.5	2		1
IFITM1	Predicted	Maternal	24.5			1	13.0			
IGF2	Imprinted	Paternal	21.8	1	2		22.1	20		4
ISM1	Predicted	Paternal	26.8	121	5	13	25.5	48	4	5
KBTBD3	Predicted	Paternal	31.8	5	1		29.5	2		1
KCNK9	Imprinted	Maternal	24.1	135	5	4	23.0	38	1	3
KCNQ1	Imprinted	Maternal	25.7	343	13	25	23.6	212	11	11
KCNQ1DN	Imprinted	Maternal	14.0	1			21.0			
KLF14	Imprinted	Maternal	21.7	2			18.4	3		
L3MBTL	Imprinted	Paternal	25.2	2			21.5	14		
LDB1	Predicted	Maternal	22.0	4			21.8	1	1	
LILRB4	Predicted	Maternal	21.4	7			21.4	7	1	
LIN28B	Imprinted	Paternal	26.9	17	1	5	27.6	48	3	4
LMX1B	Predicted	Maternal	24.0	68	8	5	24.1	53	1	3
LRRTM1	Imprinted	Paternal					15.5	1		
LY6D	Predicted	Paternal	27.3	3		1	25.0			
MAGEL2	Imprinted	Paternal					33.5	2		
MAGI2	Imprinted	Maternal	29.4	1035	78	79	27.4	1485	120	112
MEG3	Imprinted	Maternal	22.1	21	2	3	21.7	24		1
MEG8	Imprinted	Maternal	21.1	2			19.7	6		
MEST	Imprinted	Paternal	26.3	9			26.4	16		1
MESTIT1	Imprinted	Paternal	29.5	2			23.0	1		
MIMT1	Imprinted	Paternal	27.0				28.2			
MYEOV2	Predicted	Paternal	23.7	16		1	21.5			
MZF1	Predicted	Maternal	23.8	4			24.7	12	1	
NAA60	Imprinted	Maternal	24.0	10		1	23.5			
NAP1L5	Imprinted	Paternal					25.5	1		1
NDN	Imprinted	Paternal	26.0	1			22.0	1		
NKAIN3	Predicted	Paternal	27.2	539	28	35	25.9	924	69	53
NKX6-2	Predicted	Maternal	19.0							
NLRP2	Imprinted	Maternal	22.5	62	5	4	20.5	39	5	
NPAP1	Imprinted	Unknown	25.0	1		1	24.0			
NTM	Imprinted	Maternal	28.2	793	53	68	25.9	585	49	49
OBSCN	Predicted	Paternal	25.2	160	7	7	23.7	169	10	6
OR11L1	Predicted	Paternal	24.7				23.4			
OSBPL5	Imprinted	Maternal	23.8	115	9	9	22.4	89	4	4
OTX1	Predicted	Maternal	21.8	4			20.7	3		
PAOX	Predicted	Maternal	25.8	6		1	21.2	2		1
PEG10	Imprinted	Paternal	34.6	2	1		27.0	4		
PEG3	Imprinted	Paternal	25.4	1			23.7	2		
PEX10	Predicted	Maternal	19.8	8	1		19.5	12		
PHLDA2	Imprinted	Maternal					16.0	1		
PHPT1	Predicted	Maternal	21.5							
PLAGL1	Imprinted	Paternal	28.2	29	3	4	26.6	13	2	1
PPAP2C	Predicted	Maternal	18.3	16	1		26.3	276	16	26
PPP1R9A	Imprinted	Maternal	26.5	71	4	9	22.1	12	2	
PRDM16	Predicted	Paternal	21.8	231	10	17	21.7	382	10	19
PTPN14	Predicted	Maternal	27.0	10	2	3	27.3	55	4	6
PURG	Predicted	Paternal	28.2				26.8	20	3	2
PYY2	Predicted	Paternal	31.0	1						
RAB1B	Predicted	Maternal	25.1	7	2	2	23.7	3		1
RB1	Imprinted	Maternal	26.9	52	7	9	26.2	11	1	1
RBP5	Imprinted	Maternal	32.0		1	1	18.8		1	1
RPL22	Predicted	Paternal	31.9	7			26.0	1		
RTL1	Imprinted	Paternal	20.3	3			22.0	1		
SALL1	Predicted	Maternal	26.9		2	2	23.3	5	3	1
SGCE	Imprinted	Paternal	30.3	18	3	2	28.8	17	3	1
SGK2	Imprinted	Paternal	23.3	6	1	1	25.1	18	2	1
SIM2	Predicted	Paternal	26.9	28	3	2	24.2	16		1
SLC22A18	Imprinted	Maternal					22.8	29	3	
SLC22A18AS	Provisional Data	Maternal	22.3	56	4	1				
SLC22A2	Imprinted	Maternal	28.9	5		1	25.8	4		
SLC22A3	Imprinted	Maternal	27.0	82	2	8	26.8	119	2	7
SLC26A10	Predicted	Maternal	27.0	1			25.7	2		
SLC4A2	Predicted	Maternal	21.6	1			23.2	9		
SNRPN	Imprinted	Paternal	24.9	117	15	13	24.3	156	9	18
SNURF	Imprinted	Paternal	25.7	13	2	2				
SOX8	Predicted	Paternal	19.1	10			20.9	1		
SPON2	Predicted	Paternal	21.7	30		2	22.3	1		1
TCEB3C	Imprinted	Maternal	73.0	1						
TFPI2	Imprinted	Maternal					24.0			
TIGD1	Predicted	Paternal	28.4	7			25.6	7		
TMEM52	Predicted	Paternal	24.7	2		1	16.0			
TMEM60	Predicted	Paternal	29.0	3			28.3	1		
TP73	Imprinted	Maternal	23.6	73	4	5	23.0	93	3	7
TSHZ3	Predicted	Paternal	26.6	34	4	8	24.7	77	7	10
UBE3A	Imprinted	Maternal	28.3	11	1	2	26.6	105	7	10
VAX2	Predicted	Maternal	28.0	27	2	4	24.7	14	3	2
VENTX	Predicted	Maternal	25.0	1			24.0	12		
WT1	Imprinted	Paternal	28.3	53	4	2	24.9	9	1	3
ZC3H12C	Imprinted	Paternal	28.0	106	7	11	26.9	102	9	18
ZDBF2	Imprinted	Paternal	28.4	10		2	29.8	7		
ZFAT	Imprinted	Paternal	24.1	2		1	23.1	268	9	16
ZFAT-AS1	Imprinted	Paternal					28.6	7		
ZFP36L2	Predicted	Maternal	21.0	1	1		15.0	1		
ZIC1	Predicted	Maternal	37.7	2		1	30.7	2		1
ZIM2	Imprinted	Paternal	26.0			1	23.8	14		
ZNF225	Predicted	Paternal	22.1				25.4	1		
ZNF229	Predicted	Maternal	27.5	4		2	25.8	22		5
ZNF264	Unknown	Unknown	27.1	34	5	2	25.2	30	1	4
ZNF597	Imprinted	Maternal	33.0	1	1		24.8			1

For each cell line the mean depth of the alternate allele, the number of heterozygous and homozygous variants identified from WGS is provided

We have provided the examples of certain tests that could be conducted with the WGS data. We can further utilize the data generated by this platform to determine the mycoplasma contamination, presence of foreign DNA and other infectious agents as these would be present as unmapped reads. WGS analysis can be further extended to extract and annotate mitochondrial sequences, STR verification, detect the genotypes of other transplant antigens and identify plasmid/viral insertions.

## Discussion

We have shown that we can use small amounts of material to analyze samples with high content tests that yield useful results. For cGMP manufacturing, we have divided these tests into those required by regulatory authorities as part of a release criteria and tests that we classified as for information only (FIO). We have, however, argued that while not required, some subset of these tests should be incorporated into a routine testing process, as this new cell type was being considered as stating material for cell therapy for a variety of products. Our rationale for the type of tests proposed here was based on practical and theoretical consideration. Cells are intrinsically variable and can change during the manufacturing process as well as after implantation as they respond to the environment. Designing predictive tests for release is difficult in these circumstances. In addition, current models for therapy suggest that primary cells will be used where each lot will come from different starting material, or Haplobanks will be considered where multiple lines will be used to provide the same end product. We, therefore, approached this issue of comparability or equivalency by asking what factors underlie biological variability, and whether there are some simple tests that can provide data in an unbiased way that if collected in a database over time would allow us to infer what critical parameters one will need to monitor.

We proposed a transcriptome analysis, a SNP-CHIP/CGH array, and whole genome sequencing as three basic tests to complement the standard tests for pluripotency, differentiation ability and composition that are routine. It is widely agreed that karyotyping provides important information, both as an initial quality control as well as an ongoing measure of the quality of the cells, and changes in karyotype are indicative of a change in the quality of the cells. We have, however, suggested that while useful it may not be sensitive enough to predict changes in the differentiation behavior of cells or other more subtle defects that may or may not be relevant. An alternative strategy that provides additional information while requiring small amounts of material is a SNP-CHIP/CGH analysis [54, 55] where genomic DNA is hybridized to a reference genome chip set and changes across the entire genome are examined using markers ranging in number from 500 K to a million. The assay is rapid and has the advantage that additional SNP’s can be added and the chip customized as new information becomes available. Our data showed that all lines that were karyotypically normal also carried a number of deletions or duplications that included genes that are known to be altered in specific diseases or are important in their development. Some of these were identical in lines made from the same individual, indicating that they were present prior to the iPSC generation process while others were induced during the process. Although the N is small we did notice that an amplification of GNAS was present in samples from two individuals, and we suggest that monitoring this region in other lines will be important to determine if this is an anomaly or of relevance. Thus, SNP-CHIP analysis can reliably be used to monitor the karyotypic stability over time, and if a database is established then the relevant importance or frequency of a deletion or duplication can be inferred over time, so appropriate regulations on use of a line carrying a higher risk can be established.

To verify if long term cell culturing, passaging has not altered the genetic integrity of the cell lines generated and to verify the contamination issues arising due to certain laboratory practices, we also suggested to use whole genome sequencing as the WGS, in addition to confirming SNP-CHIP analysis, can be used to provide data on the state of the starting material and serve as a reference to any changes that occur in this population over time. The wealth of data is of immediate utility in providing a high-resolution map of key genes and variation between individuals that may have predictive value. In addition, as we have shown in the results, WGS provides significant additional value. We, for example, used standard software tools (BOOGIE and HLA VBSeq) to infer minor blood group antigens and high-resolution HLA typing. This information is difficult to assess without expensive tests and is not done routinely; the WGS if performed on iPSC cells could provide an initial signal as to the importance of testing recipients for particular phenotypes or minor blood group antigens.

Cross-species contamination (such as bacterial, viral or mouse) and its relative abundance in the cell lines can be estimated from the unmapped regions of the WGS data [56, 57]. Also, mitochondrial specific variants that are captured in the off-target regions are also mined from our data (unpublished data). This provides valuable information on the involvement of mitochondrial DNA in disease pathology and/or could be used as a marker to identify the individual affiliation with geography or ethnic group based on predicted mitochondrial haplogroup.

In addition to looking at any specific subset of genes that might play a role in a particular organ or cell type, one can also examine the status of imprinted genes and identify unique allelic markers for the maternal and paternal allele, allowing one to test expression of the appropriate allele in an individual. Imprinted genes are known to be important and expression from the paternal or maternal allele can have important implications. Some imprinted genes are altered during the reprogramming process, and the H/igf axis is thought to be particularly important in early development. Changes in expression can underlie a variety of developmental abnormalities and can alter the fate or quality of the differentiated cells that are obtained. Other imprinted genes are thought to be important in specific lineages. It is generally difficult to test everything in every line but by having the WGS of the line in a database, one can imagine inferring the phenotype when required by comparing the genomic sequence with RNA Seq performed at an appropriate time. In particular, one may be able to determine if the X chromosome is randomly inactivated or not as a complement to the transcriptome analysis.

The WGS data can also be queried to cross examine the SNP’s present or absent in the actual sample to provide better clarity on the changes seen in CGH/SNP chip analysis, as lack of hybridization could be due to absence of the SNP or allelic variability. We have shown (unpublished data) that a virtual SNP-CHIP result can be generated from WGS and a subset from exome sequencing SNP’s. This may be more relevant for ethnic subgroups and can be used as an independent verification of the SNP-CHIP result. The importance of such an analysis is highlighted by the lack of overlap seen between the WGS and SNP/CHIP hybridization results (see results). Others have made similar observations. Rogers and colleagues [58] have evaluated on the levels of discordance across sequencing, imputation and microarray platforms and reported that the most common type of discordance comes from missing genotypes on the sequence technology, which occurred most frequently when the microarray technology identified at least 1 reference allele at the variant site.

We show that array-based transcritpome analysis can be used to assess overall interchangeability of the starting population using a R2 correlation co-efficient or comparing the profile with currently available databases such as Pluritest [59, 60]. The RNA seq data, in addition, provides a complement to the more specific tests that are performed as standard quality control and also allows us to go back and ask additional questions as to the quality of the cells that one may not ask routinely. For example, while we examine pluripotency using a couple of markers we could easily use the array-based results to identify another 150 potential pluripotency markers, or reexamine if trophectoderm is a contaminant, or some cells have already acquired a epiblast fate, or for distinguishing between naïve and primed states. Having the transcriptome analysis in a database may also allow one to assess batch-to-batch variability and define specs for any release criteria one may establish.

We chose to use transcriptome analysis, SNP-CHIP and exome sequencing rather than alternatives, because we felt that these tests provide sufficient information and represent the best cost-benefit compromise. These tests are widely available, can be done worldwide and the analysis pipeline is relatively standard. The tests provide several internal controls and the data can be readily cross-correlated. Furthermore, database repositories that are geared to receiving such data-sets have been established in multiple countries. We have also suggested that while the tests themselves are of utility as internal controls for any organization, we feel the tests could be more widely applicable for comparisons across sites and groups, provided uniformity in data collection and storage was in place and comparators or calibration material was available. In principle, any line can be used as long as it is widely available, stable and well characterized. We have proposed that a set of ten lines that we generated at the NIH, which are widely disseminated and are well characterized and have been grown in different culture conditions and have been shown to differentiate into the major phenotypes of interest, could serve as such calibration material. The NIH has generated whole genome sequencing data and in association with RUDCR (Rutgers) SNP-CHP/CGH and RNA-SEQ data, and as such, these lines could serve such a purpose.

It is important to emphasize that these tests are by no means an absolute standard, and certainly do not enable complete characterization of lines. For example, one can miss many epigenetic changes that may be important, or one may miss point mutations or inversions given the depth and resolution of the analysis we have chosen. We feel, however, these tests are a critical first step in developing a database of information that can offer a path to developing cheaper, more focused tests, as information on the lines evolves and provides a path to more rigorous testing, at a time when costs for more detailed testing decrease.

In summary, we have shown that for a relatively modest investment useful additional information can be obtained, and this information if shared widely and accumulated in a database along with data from calibration material will allow us to assess the safety of therapy and provide regulators with a strategy to assess Haplobanks and other models for delivering cell-based therapy.

## Electronic supplementary material

Below is the link to the electronic supplementary material.

Supplemental Table 4 (XLSX 69 kb)
